# Orthogonal upconversion supramolecular microneedles promote endogenous ferroptosis in keloids

**DOI:** 10.7150/thno.108289

**Published:** 2025-05-08

**Authors:** Wenchang Lv, Yue Zhang, Yiping Wu, Hongbo Chen

**Affiliations:** 1 Department of Plastic and Cosmetic Surgery, Tongji Hospital, Tongji Medical College, Huazhong University of Science and Technology, Wuhan 430074, China; 2 Key Lab of Material Chemistry for Energy Conversion and Storage, Ministry of Education, and Hubei Key Lab of Materials Chemistry and Service Failure, School of Chemistry and Chemical Engineering, Huazhong University of Science and Technology (HUST), Wuhan 430074, China

**Keywords:** orthogonal upconversion supramolecular microneedle, endogenous ferroptosis, photodynamic therapy, synergistic therapy, keloid

## Abstract

**Background:** Keloids represent a type of tumor-like fibroproliferative disease, which can not only cause aesthetic damage but also threaten health. Current therapies often lack precision and efficacy, necessitating minimally invasive and targeted strategies.

**Methods:** This study developed orthogonal upconversion supramolecular microneedles (OUSMNs) integrated with surface-functionalized upconversion nanoparticles (UCNPs) for intelligent keloid therapy. The UCNPs were functionalized by the ferritin homing peptide (HKN_15_) and a photosensitizer (rose Bengal), which could target keloid fibroblast (KF) and generate singlet oxygen (^1^O_2_), thereby inducing endogenous ferroptosis. Mechanistic effects on PI3K-AKT, mTOR, and ferroptosis pathways were analyzed by transcriptome analysis and rescue experiments.

**Results:** The OUSMNs are strong and tough to effectively penetrate the fibroproliferative tissue, and can rapidly dissolve in keloids within 60 s, which makes the UCNPs easy to target the KF by the ferritin-homing peptide HKN_15_ on the particle surface. The targeting process can be tracked by the red-color upconversion emission under 980 nm laser. On the other hand, upon 808 nm laser irradiation, the UCNPs can lead to the generation of ^1^O_2_. The ^1^O_2_ not only result in endogenous ferroptosis by destroying the ferritin, but also give rise to synergistic photodynamic therapy that can effectively combat keloids through inhibiting the PI3K-AKT and mTOR pathways while activating the ferroptosis pathway.

**Conclusions:** The proposed OUSMNs promise practical applications for minimally invasive, precise and intelligent keloid therapy.

## Introduction

Abnormal skin scaring has significantly threatened the health of hundreds of millions of patients, forcing us to develop timely and effective treatment methods 1-3. Keloids, which are tumor-like scars with fibroproliferative and exophytic outgrowing characteristics, can not only cause pruritus and pain but also lead to aesthetic damage and dysfunction 4. They are easy to occur by abnormally wound healing after injury and even a minor trauma or infection, which however are very difficult to treat and ready to recur due to the unclear etiology and pathogenesis 5, 6. Hereto, treatment methods such as surgical excision 7, radiotherapy 8, corticosteroid injections 9, laser therapy 10, cryotherapy 11, and chemotherapy 12, have limited success. For example, the recurrence rate after surgical excision is reported to be over 50% 13. The low clinical curative ratio and high recurrence rate have imposed a great burden on both the economy and society, loading urgent demands for new methods to treat keloids.

Inhibiting cell proliferation is key to combat keloids, for which ferroptosis has been recognized as valuable 14. Ferroptosis represents non-apoptotic cell death that is discovered by Stockwell in 2012 and characteristic with iron-induced accumulation of lipid peroxides (LPO) 15. Excessive LPO resulting from the iron-involved Fenton reaction have been proven effective to disrupt cell membrane 16. Therefore, exogenous iron supplementation has been extensively exerted both *in vitro* and *in vivo* by inducing iron-based nanomaterials 17. However, exogenous iron supplementation can pose potential health risks, such as immunotoxicity 18 and neurotoxicity 19, which limits practical applications in clinic. In contrast, endogenous ferroptosis exhibits an enhanced safety profile and has demonstrated therapeutic efficacy in selectively eliminating breast tumor cells. This approach leverages tumor-targeting agents (e.g., Ce6-PEG-HKN_15_) to disrupt intracellular ferritin in proliferating cells, thereby triggering iron-mediated cytotoxicity by releasing iron ions 20. Ferritin, a spherical protein complex comprising an outer apoferritin shell and an inner mineral core that stores 4500-5000 iron atoms 21, can initiate the Fenton reaction through iron liberation during its degradation. Notably, elevated ferritin levels have been identified in hyperproliferative keloid fibroblasts (KFs) 22, suggesting these pathological cells maintain substantial iron reservoirs capable of triggering endogenous ferroptosis. This iron reserving mechanism primarily stems from dual metabolic reprogramming in KFs: enhanced cellular iron uptake and storage capacity concurrent with impaired iron efflux pathways during their aberrant growth phase 23. However, while ferroptosis induction has demonstrated therapeutic efficacy in breast cancer models, its endogenous activation in keloid pathology remains poorly characterized. Current strategies for initiating endogenous ferroptosis rely predominantly on systemic intravenous agents and red-color light triggering 20, approaches inherently limited by suboptimal biodistribution and hepatic first-pass metabolism. Notably, the dense extracellular matrix of keloid tissue further exacerbates drug delivery challenges. These limitations underscore the critical need for developing localized therapeutic platforms capable of precise spatiotemporal control and direct intralesional cargo delivery.

Microneedle (MN) drug delivering has garnered extensive attention in recent years as it can directly release drugs to local lesion sites with minimal invasion 24. MNs must simultaneously achieve two critical design objectives: mechanical strength sufficient to breach biological barriers and controlled dissolution rates for timely drug release. Current polymer-based MNs typically employ non-crystalline, water-soluble macromolecules to balance these requirements 25. However, the polymer MNs are difficult to rapidly dissolve in dense keloid tissues despite their sufficient mechanical strength. The slow dissolving process prolongs wear time of patches and delays the onset of payload action 26. Emerging supramolecular MN designs address this limitation through small molecules that self-assemble via reversible noncovalent bonds 27, and exhibit accelerated dissolution profiles while maintaining or even surpassing the mechanical performance of polymeric counterparts 28. However, a critical technological gap persists in current supramolecular MN platforms: the absence of integrated laser-responsive functionality for real-time therapeutic monitoring and on-demand treatment activation 29, 30. This limitation fundamentally hinders closed-loop control of drug pharmacokinetics in complex pathological microenvironments.

**Scheme 1:**
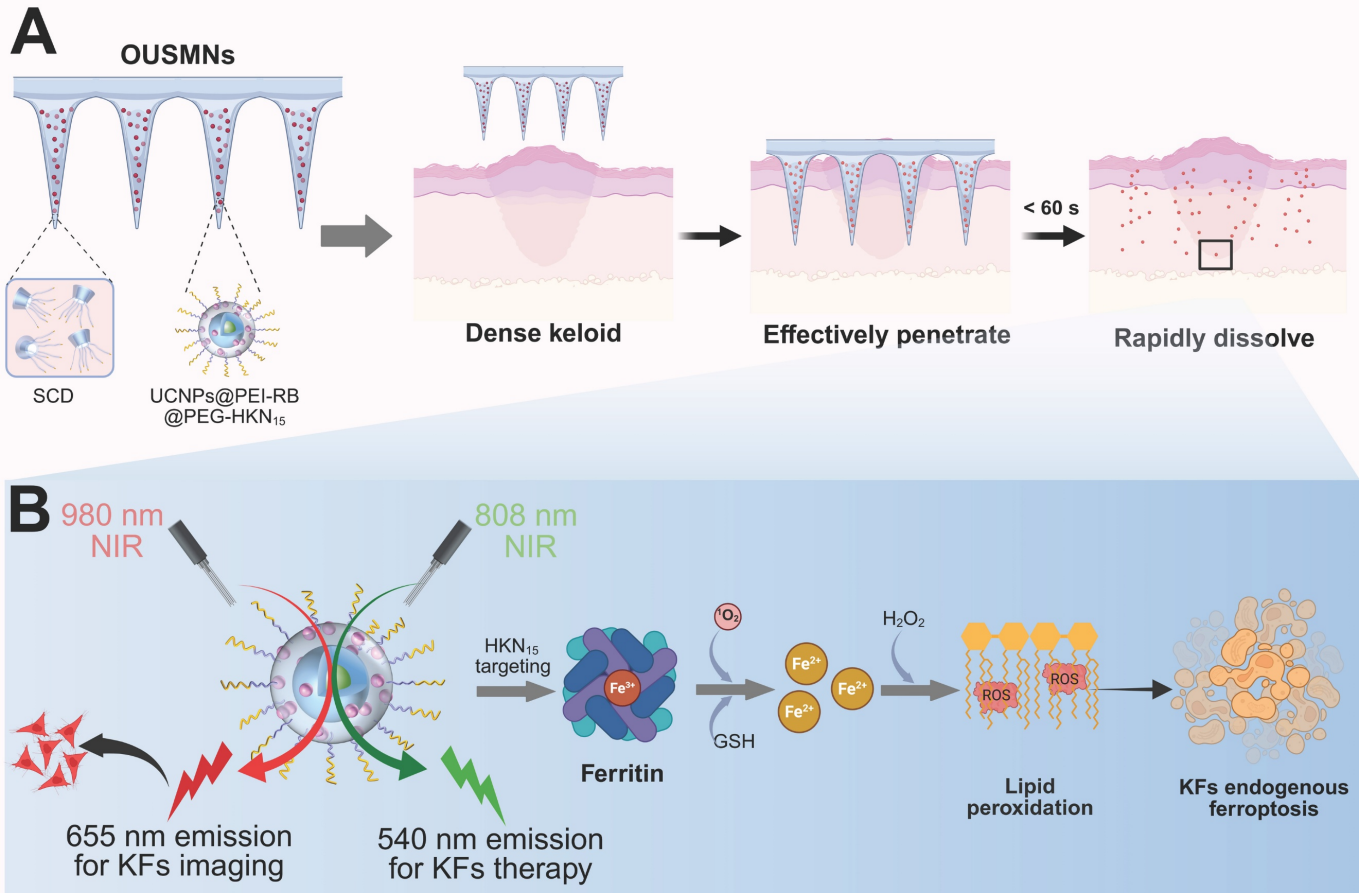
Proposed OUSMNs for keloid therapy. (A) Schematic illustration of the design and application of OUSMNs in keloids. (B) The therapeutic mechanisms of UCNPs@PEI-RB@PEG-HKN_15_ for the endogenous ferroptosis and synergistic photodynamic therapy (PDT) in keloids.

To tackle the above challenges, we herein design a novel platform of orthogonal upconversion supramolecular microneedles (OUSMNs). This design is built on the basis of supramolecular microneedles reported by Zhang and Zhu 28. As illustrated in **Scheme 1**, OUSMNs are basically composed of two types of components, namely, supramolecular sulfobutylether-*β*-cyclodextrin (SCD), and surface-functionalized upconversion nanoparticles (UCNPs) with ferritin-homing peptide HKN_15_ and rose Bengal (RB). This novel platform (OUSMNs) possessing synergistic therapy, has displayed three main advantages for keloid treatments: (1) OUSMNs can not only effectively penetrate dense keloid tissues but also fast dissolve within 60 s for efficiently delivering payload UCNPs in the deep dermal layers; (2) The UCNPs precisely approach the lesion sites with the assistance of targeting peptide HKN_15_; (3) The UCNPs exhibit orthogonal emissions under 808 nm and 980 nm lasers, respectively, allowing for real-time imaging of the keloid treatment process. The targeting process of UCNPs to KFs can be monitored in a thickness up to 0.5 mm within 4 h by virtue of 655 nm upconversion emission under 980 nm near-infrared (NIR) laser, and the endogenous ferroptosis in KFs can be triggered using the 540 nm upconversion emission under 808 nm NIR laser. Collectively, this platform OUSMNs could trigger the drug acting at any time once the keloid lesion sites are successfully targeted, consequently leading to improved treatment efficacy. This platform OUSMNs offer a valuable angel for combating keloids based on endogenous ferroptosis induction.

## Methods

### Materials

Erbium(III) chloride hexahydrate (ErCl_3_·6H_2_O), ytterbium(III) chloride hexahydrate (YbCl_3_·6H_2_O), thulium (III) chloride hexahydrate (TmCl_3_·6H_2_O), oleic acid (OA), 1-octadecene (ODE), tetrahydrofuran (THF), n-hexane, ethanol, diethyl ether, N,N-dimethylformamide (DMF), dimethyl sulfoxide (DMSO), rose bengal (RB), 1-ethyl-(3-dimethylaminopropyl) carbodiimide hydrochloride (EDC∙HCl), N-hydroxysuccinimide (NHS), nitrosonium tetrafluoroborate (NOBF_4_), and ammonium fluoride (NH_4_F) were all procured from Beijing Innochem Technology Co., Ltd. Ferritin-targeting polypeptide (HKNKGKKNGKHNGWK; HKN_15_) was synthesized by Apeptide Co., Ltd (Shanghai, China). Branched polyethyleneimine (PEI, 10 kDa), glutathione (GSH), and 1,3-diphenylisobenzofuran (DPBF) were obtained from Sigma-Aldrich (USA). DMEM/F12 medium, fetal bovine serum (FBS), phosphate-buffered saline (PBS), and trypsin were sourced from Gibco (USA). Calcein-AM/PI dual fluorescence staining kit was purchased from Yeasen Biotechnology Co., Ltd. (Shanghai, China). The BODIPY^581/591^-C11 probe and 3'-(p-hydroxyphenyl) fluorescein (HPF) probe were obtained from Thermo Fisher Scientific (USA). Dialysis bags used in the experiments were purchased from Spectrum Laboratories.

### Preparation of surface-functionalized UCNPs

The synthesis of surface-functionalized upconversion nanoparticles (UCNPs@PEI-RB@PEG-HKN_15_) consisted of three successive steps.

#### Preparation of orthogonal UCNPs

Orthogonal UCNPs with a core-shell structure were synthesized using an enhanced thermal co-precipitation method. The synthesized orthogonal UCNPs were collected via centrifugation and washed with ethanol to remove any residual impurities and reactants. Finally, the cleaned orthogonal UCNPs were dispersed in cyclohexane for subsequent applications and research.

#### Preparation of PEI-RB

Synthesis of RB-COOH: RB (1 mmol), 6-bromohexanoic acid (1.2 mmol), and 20 mL of acetone/water (7:3 v/v) were combined in a 50 mL round-bottom flask. The mixture was heated to 75 °C under an argon atmosphere and reacted for 24 h. After completion, the acetone and water were removed by rotary evaporation. The crude product was washed three times with water and diethyl ether, then freeze-dried to yield RB-COOH.

Synthesis of PEI-RB: RB-COOH (30 mg, 0.03 mmol), EDC (30 mg, 0.15 mmol), and NHS (30 mg, 0.65 mmol) were added to a 50 mL round-bottom flask with 20 mL of DMF and stirred at room temperature for 2 h. PEI (10 kDa, 300 mg, 0.03 mmol) was then added, and the mixture was stirred vigorously for 24 h. The solvent was removed by rotary evaporation to yield the crude product, which was purified by dialysis and characterized by ^1^H NMR and FT-IR.

#### Preparation of NHS-PEG-HKN_15_

NHS-PEG-HKN_15_ was synthesized via a click chemistry reaction between a thiol (SH) and a maleimide (MAL), where covalent bonding occurs through nucleophilic addition. Briefly, HKN_15_-SH (0.01 mmol) and MAL-PEG-NHS (0.01 mmol) were dissolved in 5 mL of DMSO. The reaction mixture was stirred at room temperature for 6 h, followed by dialysis in distilled water for 48 h. The final product, NHS-PEG-HKN_15_, was obtained by lyophilization, and then characterized by ^1^H NMR and HRMS mass.

In summary, UCNPs@PEI-RB was first prepared by ligand exchange, and then UCNPs@PEI-RB@PEG-HKN_15_ was prepared by amidation reaction.

### Fabrication of OUSMNs

Preparation of matrix solution: 1.5 g of SCD was dissolved in 2 mL of various concentrations of surface-functionalized UCNPs aqueous solution with magnetic stirring at room temperature, forming a homogeneous and transparent matrix solution (150% w/v).

Preparation of OUSMNs were fabricated using a straightforward vacuum-assisted molding technique. Briefly, various concentrations of the supramolecular nano-matrix solution were poured into PDMS molds, vacuumed to -0.08 MPa, and left to stand for 10 min. The molds containing the matrix solution were then placed in a desiccator with a desiccant and dried at room temperature for 24 h. Once dried, the OUSMNs were peeled off from the flexible PDMS molds.

### Primary fibroblasts were extracted and cultured

The keloid and normal skin tissues collected were placed in centrifuge tubes with PBS to prevent dehydration. After removing the epidermis using trypsin, the dermal tissues were finely minced with sterile scissors. Collagenase was then added to digest the tissues and release fibroblasts. The cell suspension was filtered to remove residual tissue fragments and centrifuged to pellet the cells. These cells were resuspended in DMEM/F12 medium with 10% fetal bovine serum and 1% antibiotics, then seeded into culture flasks. The flasks were incubated at 37 °C and 5% CO_2_ to culture primary fibroblasts. This study received approval from the ethics committee of Tongji Hospital Affiliated to Huazhong University of Science and Technology, and informed consent was obtained from all patients providing tissue samples.

### CCK-8 assay

The CCK-8 assay was widely used to assess cell proliferation and cytotoxicity. Firstly, 100 μL of cell suspension was seeded into a 96-well plate at a density of 1.0 ×10^4^ cells/mL and incubated at 37 °C with 5% CO_2_ for 12 h. KFs were then co-incubated with PBS (control, Group I), SCD (Group II), UCNPs@PEI-RB&SCD (Group III), UCNPs@PEG-HKN_15_&SCD (Group IV), and UCNPs@PEI-RB@PEG-HKN_15_&SCD (Group V) for 4 hours, followed by irradiation with 808 nm NIR. In biological experiments, the 808 nm laser parameters were optimized to 0.83 W/cm² with an intermittent irradiation protocol (20-min total duration: 1-min irradiation followed by 3-min intervals) to ensure both therapeutic efficacy and biosafety. Each well was then replaced with 100 μL of fresh medium, and 10 μL of CCK-8 solution was added, followed by incubation in the dark for 2 h to allow the CCK-8 reagent to react with live cells. Absorbance was measured using a multi-mode microplate reader at 450 nm.

### Evaluation of intracellular iron ion and ·OH content

iron ion content: The CAL-AM method indirectly evaluates cytoplasmic iron ion levels. Membrane-permeable CAL-AM quickly enters the cytoplasm and releases the fluorescent compound CAL. In the presence of iron ions, CAL binds to free iron in the cytoplasm, resulting in fluorescence quenching. Thus, CAL fluorescence intensity was inversely correlated with intracellular iron ion levels. KFs were co-incubated with various surface-functionalized UCNPs in 6-well plates for 4 h. After PBS washing, the cells were irradiated with an 808 nm laser for 20 min, followed by incubation in serum-free medium containing CAL-AM (0.2 μM) for 15 min. The cells were then collected, resuspended in PBS (400 μL), and analyzed using flow cytometry. The average fluorescence intensity of each group inversely correlates with the intracellular iron ion levels.

·OH content: HPF was a non-fluorescent molecule that becomes highly fluorescent upon reacting with ·OH, converting into fluorescein. After treatment with different surface-functionalized UCNPs, the cells were irradiated with an 808 nm laserfor 20 min. Subsequently, KFs were incubated in fresh medium containing HPF (20 μM) for 30 min, and then imaged using a CLSM.

### Mitochondrial membrane potential detection

5,5',6,6'-tetrachloro-1,1',3,3'-tetraethylbenzimidazolylcarbocyanine iodide (JC-1) was a fluorescent dye widely used in cellular studies to evaluate changes in mitochondrial membrane potential. KFs were treated as previously described. Adding 500 μL of JC-1 working solution to the dishes and incubate at 37 °C in the dark for 20 min to allow JC-1 to penetrate the cells and localize in the mitochondria. JC-1 aggregates under high mitochondrial membrane potential, emitting red fluorescence. Under low mitochondrial membrane potential, JC-1 remains monomeric, emitting green fluorescence. Normally functioning mitochondria will primarily show red fluorescence, while impaired mitochondria with reduced membrane potential will show green fluorescence. By measuring the intensity of red and green fluorescence, changes in mitochondrial membrane potential can be quantified.

### Migration and invasion assessment

Wound healing assay: KFs (7.0 × 10^5^ cells/well) were seeded in 6-well plates and cultured for 24 h to form a confluent monolayer. A uniform scratch was made using a 200 μL pipette tip. The wells are washed with PBS to remove detached cells, ensuring that the scratch is filled only by migrating cells. Cell migration and scratch closure were monitored with a microscope, and the scratch width is recorded at 0 and 24 hours.

Transwell assay: The Transwell assay was commonly used to study cell migration and invasion. For the migration assay, 2.0 × 10^4^ cells in serum-free medium were seeded into the upper chamber of a Transwell insert (8 μm pore size, Corning). For the invasion assay, the upper chamber is pre-coated with Matrigel (Corning) before seeding the cells. Medium with 20% FBS was added to the lower chamber. After incubating at 37 °C for 24 h, cells were fixed with 4% paraformaldehyde for 20 min and stained with 0.2% crystal violet for 15 min. Non-migrated cells in the upper chamber are removed with a cotton swab. The migrated or invaded cells in the lower chamber were observed and counted under an optical microscope.

### LPO detection

C11-BODIPY^581/591^ was a fluorescent probe used to monitor LPO levels in cells in real-time quantitatively. This lipophilic probe, once oxidized by intracellular LPO, shifts its fluorescence emission from red (λex: 581 nm, λem: 591 nm) to green (λex: 488 nm, λem: 510 nm). First, KFs were seeded in confocal dishes at 2.0 × 10^4^ cells/well and cultured for 12 h. After treatment with various surface-functionalized UCNPs, the cells were irradiated with an 808 nm laser, then incubated with fresh DMEM/F12 medium containing C11-BODIPY^581/591^ (5 μM) for 20 min. Finally, the cells are washed with PBS and imaged using a CLSM.

Malondialdehyde (MDA) was a lipid peroxidation product frequently used as a biomarker to evaluate oxidative stress levels. A common detection method was the TBARS (thiobarbituric acid reactive substances) assay, which involved thiobarbituric acid (TBA) under acidic conditions to produce a stable colored product. KFs were treated as previously described. 100 μL of cell lysate was mixed with TBA reagent and boiled for 60 min. After cooling, the supernatant's absorbance was measured at 532 nm. Concurrently, the protein concentration was determined using a BCA protein assay kit to normalize the MDA levels.

### Cell membrane integrity detection

3,3'-dioctadecyloxacarbocyanine (DiO) was a lipophilic fluorescent dye commonly used to stain cell membranes and other lipid-soluble biological structures. It rapidly diffused into the cell membrane, providing uniform staining. To prepare a 2 mM stock solution, dissolve 10 mg of DiO powder in DMSO. Dilute this stock solution with serum-free medium to make a 2 μM working solution for cell experiments. Briefly, seed KFs into confocal dishes at a density of 2.0 × 10^4^ cells/well and culture for 12 h. After various treatments, replace the medium with fresh medium containing DiO dye. Incubate for 20 min to ensure the DiO dye fully integrates into the phospholipid bilayer of the cell membrane. Wash the cells with serum-free medium to remove any unbound DiO dye. Using a CLSM to observe the cells and detect DiO fluorescence signals. Intact cell membranes will exhibit strong green fluorescence, while damaged membranes will show a reduced or absent signal. Flow cytometry can also be used to quantitatively analyze the cells, measuring DiO fluorescence intensity to assess cell membrane integrity and damage.

### Subcutaneous transplantation model of nude mice with keloid

This study used female BALB/c nude mice to create a patient-derived keloid graft model, adhering to the institutional ethics committee guidelines. Briefly, fresh keloid specimens (within 4 h post-surgery) were de-epidermized and cut into uniform blocks approximately 9×8×7 mm³ using tissue scissors. These tissue blocks were implanted into the upper back, lower back, and abdomen of 6-week-old female BALB/c nude mice. To prevent drug diffusion from affecting results, implantation sites were spaced at least 3 cm apart. Each keloid tissue block was weighed using a high-precision digital milligram scale before implantation. Interventions were initiated approximately one week after implantation, after the sutures had fallen off. PBS was used as the injection medium, standardized to 0.1 mL per site. Interventions were administered every 2 days for a total of 5 times. Ten days after the final intervention, the mice were sacrificed, and the keloid grafts were collected. Each tissue sample underwent weight and volume analysis, immunohistochemical staining, and hematoxylin and eosin staining.

### Biosafety assessment

To evaluate the potential toxicity of various surface-functionalized UCNPs, the body weight of the mice was monitored daily during the treatment. After the intervention, major organs (heart, liver, spleen, lung, kidney) and blood samples were collected from each group of mice for H&E staining and blood biochemical analysis, respectively.

### Statistical analysis

Two groups were compared using Student's t-test and multiple groups were compared using one-way analysis of variance (ANOVA). The significant difference was defined as *P < 0.05, **P < 0.01, or ***P < 0.001.

## Results and Discussion

### Synthesis and Surface-functionalization of Orthogonal UCNPs

Orthogonal UCNPs in the core-shell structure and hexagonal phase were first synthesized via a modified method 31. The core was NaErF_4_:Tm^3+^/Yb^3+^ (Er^3+^/Tm^3+^/Yb^3+^:80.0/0.5/19.5 mol) and the shell was NaYbF_4_, which was able to generate red and green color emissions without crosstalk under 980 and 808 nm lasers, respectively.

**Figure 1:**
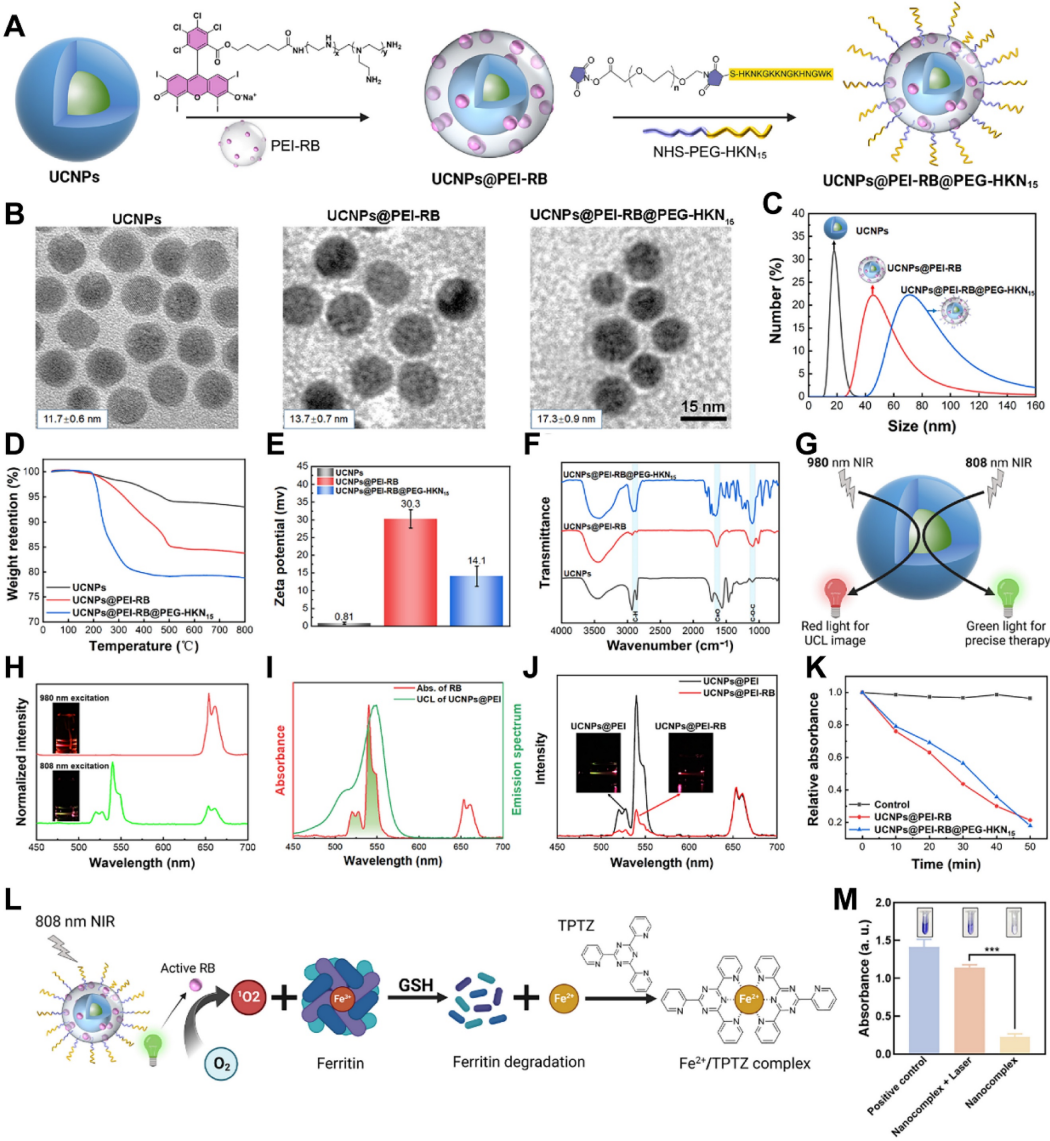
** Synthesis and surface-functionalization of orthogonal UCNPs**. (A) Preparation diagram, (B) TEM images, (C) Dynamic light scattering results, (D) thermal gravimetric analysis, (E) Zeta potential, and (F) Fourier-transform infrared spectroscopy of UCNPs, UCNPs@PEI-RB, and UCNPs@PEI-RB@PEG-HKN_15_. (G) Diagram illustrating orthogonal functions of UCNPs. (H) Upconversion luminescence (UCL) spectrum and emission picture of orthogonal UCNPs. (I) UCL spectra of the UCNPs under 808 nm NIR excitation and UV-visible absorption spectra of RB. (J) UCL spectra of UCNP@PEI-RB and UCNP@PEI under 808 nm NIR light irradiation. (K) Absorption attenuation curves of DPBF at 410 nm against time under 808 nm NIR radiation. (L) Schematic diagram illustrating ferritin degradation and iron detection principle. (M) The digital image and quantitative analysis of iron content, wherein data were given as mean ± SD, n = 3. **P < 0.01, ***P < 0.001, ****P < 0.0001.

To enhance the water solubility, photosensitivity, and targeting functions, we implemented surface functionalization of the orthogonal UCNPs using PEI-RB and NHS-PEG-HKN_15_ (**Figure 1A**). PEI and PEG stood for poly (ethylene imine) and poly (ethylene glycol), respectively, which showed good water solubility. PEI-RB was synthesized via the amidation of PEI with RB-COOH, which could be further modified by NHS-PEG-HKN_15_ using the residual amine groups. NHS-PEG-HKN_15_ was synthesized via the thiol-maleimide click reaction with mercapto-terminated HKN_15_ and maleimide-terminated NHS-PEG as the reactants, where NHS denoted *N*-hydroxysuccinimide. The structure of the synthesized chemicals was verified by proton nuclear magnetic resonance, high-resolution mass spectrometry, and Fourier-transform infrared spectroscopy (**Figure S2-S11**).

The success of surface-functionalization of the orthogonal UCNPs with PEI-RB and PEG-HKN_15_ was comprehensively verified using transmission electron microscopy (TEM), dynamic light scattering (DLS), thermal gravimetric analysis, Zeta potential, and infrared spectroscopy. TEM analysis in **Figure 1B** revealed uniform dimensions of the synthesized UCNPs, UCNPs@PEI-RB, and UCNPs@PEI-RB@PEG-HKN_15_, whose diameter increased gradually from 11.7 ± 0.6 to 13.7 ± 0.7 and 17.3 ± 0.9 nm, respectively. The surface-functionalization led to a dramatic polarity change of UCNPs so that the cyclohexane dispersible nanoparticles transformed into water dispersible. DLS analysis confirmed the dimension increase by sequential surface-coating. Notably, the measured sizes were larger than in TEM due to the solvation effect (**Figure 1C**). Infrared spectroscopy further confirmed the surface modification of UCNPs. As displayed in **Figure 1D**, UCNPs@PEI-RB showed a characteristic peak at 1653 cm^-1^ (C=O stretching vibration), while UCNPs@PEI-RB@PEG-HKN_15_ exhibited characteristic peaks at 1653 cm^-1^ (C=O stretching vibration), and 1093 cm^-1^ (C─O─C stretching vibration). Due to surface-coating, larger weight loss was observed when heated to 800 °C, e.g., 8%, 20%, and 24% for UCNPs, UCNPs@PEI-RB, and UCNPs@PEI-RB@PEG-HKN_15_, respectively (**Figure 1E**). However, the zeta potential first increased from +0.81 to +30.3 mV and then decreased to +14.1 mV (**Figure 1F**). According to the UV-vis absorption, the RB content in UCNPs@PEI-RB@PEG-HKN_15_ was approximately 7.35 wt.% (**Figure S13**).

The orthogonal UCNPs generated green-color emission peaked at 540 nm and red-color emission at 655 nm when excited by 808 and 980 nm NIR light, respectively (**Figure 1G-H**). RB was selected as the photosensitizer due to its absorption spectrum closely aligning with the upconversion peak emission at 540 nm (**Figure 1I**), promising NIR PDT 32. Under 808 nm excitation, the green-color emission of UCNP@PEI-RB was remarkably reduced compared to UCNP@PEI without RB, which was ascribed to energy transfer from UCNPs to RB (**Figure 1J**). The excitation of RB could generate singlet oxygen (^1^O_2_). The indicator was used 1,3-Diphenylisobenzofuran (DPBF) to detect ^1^O_2_ production 33. Once oxidized by ^1^O_2_, DPBF showed altered absorption peak. **Figure S12** illustrated the absorption spectra of DPBF in UCNPs@PEI-RB and UCNPs@PEI-RB@PEG-HKN_15_ solutions upon 808 nm NIR irradiation. The results demonstrated that, compared to PBS control group, the absorbance intensity of UCNPs@PEI-RB@PEG-HKN_15_ at 410 nm gradually decreased with prolonged 808 nm NIR irradiation time, indicating the effective generation of ^1^O_2_ (**Figure 1K**).

The key trigger for endogenous ferroptosis was that ^1^O_2_ produced by surface-functionalized UCNPs could effectively destroy ferritin. We evaluated the photodynamic disruption of ferritin using the iron indicator 2,4,6-tripyridyl-s-triazine (TPTZ) (**Figure 1L**). As reported, under acidic conditions, the binding of Fe³⁺ in horse spleen ferritin (HSF) is disrupted, leading to the release of Fe³⁺ 34. The addition of a reducing agent, such as glutathione (GSH), converts Fe³⁺ into Fe²⁺, which is more stable and detectable. TPTZ then forms a deep blue complex with Fe²⁺, with its absorbance directly correlating with the concentration of Fe²⁺. The results showed that, compared to the nonirradiated group, the UCNPs@PEI-RB@PEG-HKN_15_ solution formed a distinct deep blue complex after 808 nm NIR irradiation (**Figure 1M**), indicating the significant release of Fe²⁺. This observation was further confirmed by SDS-PAGE analysis (**Figure S14**), which showed a similar trend, supporting the conclusion that the surface-functionalized UCNPs effectively disrupted ferritin and released iron upon NIR irradiation.

### Synthesis and Characterization of OUSMNs

**Figure 2:**
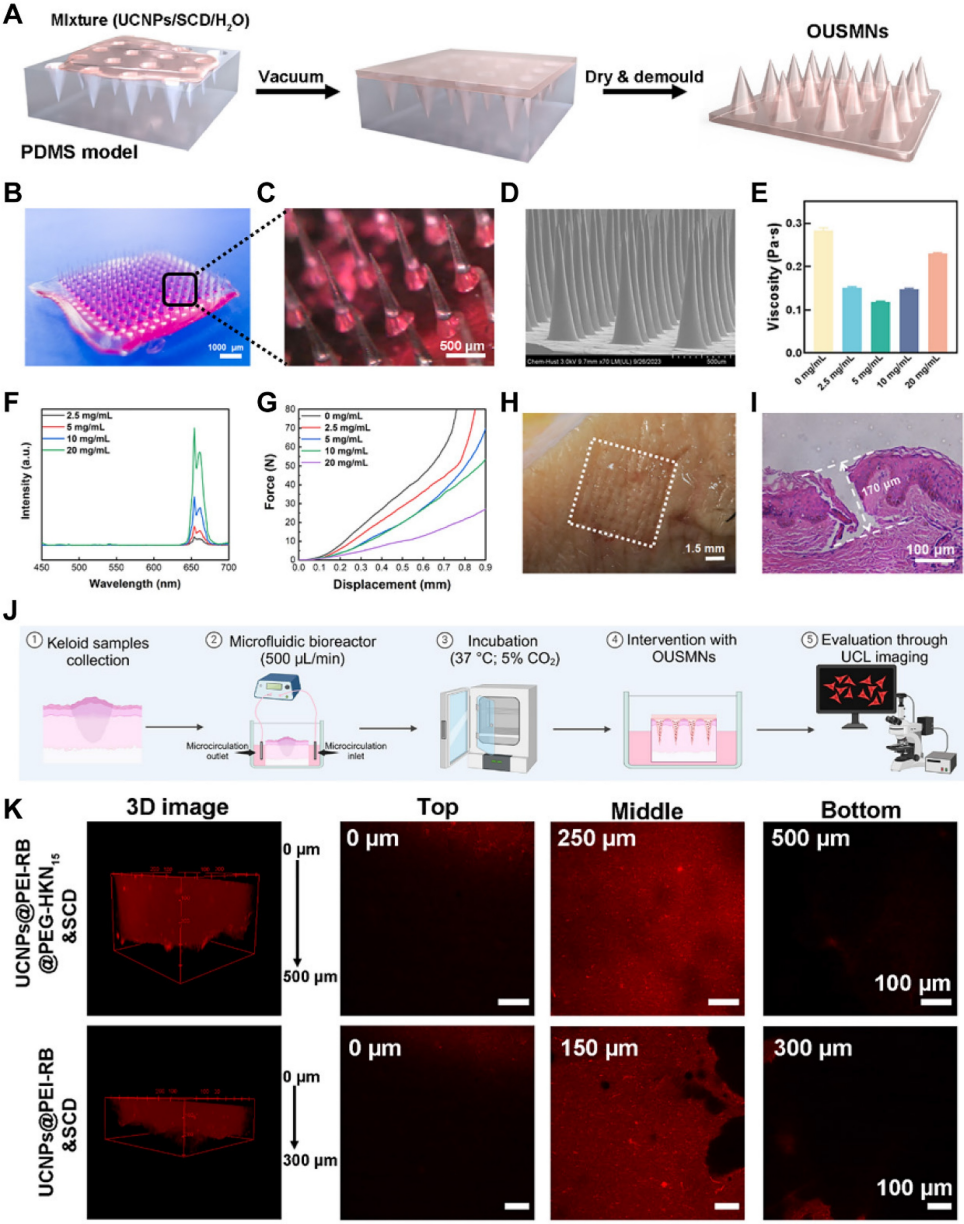
Synthesis and characterization of OUSMNs. (A) The preparation process, (B, C) macroscopic, and (D) SEM images of OUSMNs. (E) Precursor viscosity in water, (F) UCL spectra and (G) compressive force-displacement curves of OUSMNs at different concentrations. (H) Picture of OUSMNs-treated keloid. (I) Optical microscopy images showing cross-sections of keloids treated with OUSMNs. (J) Diagram depicting the culture of *ex vivo* keloid tissue in a microfluidic bioreactor. (K) The diffusion of UCNPs (with or without HKN_15_ on the particle surface) from OUSMNs within *ex vivo* keloid tissue.

The most prominent advantage of MN-assisted drug delivery is the ability to painlessly penetrate the skin, enhancing the penetration efficiency of therapeutic drugs 25. This method is more acceptable to patients and makes self-administration more feasible. We successfully prepared OUSMNs using a low vacuum-assisted molding technology (**Figure 2A**). After demolding and drying, we obtained OUSMNs with a well-structured morphology and transparent appearance (**Figure 2B-C**). Meanwhile, scanning electron microscopy (SEM) images showed that the prepared conical OUSMNs had a height of approximately 900 µm, a tip-to-tip distance of approximately 550 µm, and a base width of approximately 350 µm (**Figure 2D and Figure S15**).

Rheological analysis revealed that at a concentration of 5 mg/mL of surface-functionalized UCNPs, the viscosity of the precursor in water dropped to its minimum, namely, 0.115 Pa·s (**Figure 2E**). In contrast, the widely used polymer MN precursor in water exhibited relatively higher viscosities, e.g., 0.3-2.0 Pa·s (**Figure S16**). As reported, the viscosity of matrix solution dictated the dissolution rate of MNs and the diffusion efficiency of the loaded drug 35. Benefiting from the low viscosity, the OUSMNs exhibited a fast dissolution rate (< 30 s) in a gelatin skin model (**Figure S17**). Besides, the UCL intensity of OUSMNs was positively correlated with the concentration of surface-functionalized UCNPs (**Figure 2F**), thereby enabling UCL-guided targeted drug delivery and real-time monitoring of drug diffusion.

The OUSMNs demonstrated excellent mechanical strength, which was crucial for penetrating dense keloid tissues. As shown in **Figure 2G and Figure S18**, even though the addition of UCNPs slightly reduced the mechanical strength of OUSMNs. Our designed OUSMNs still outperformed commonly used soluble polymer MNs reported in the literature 36, 37. Notably, OUSMNs at various concentrations did not exhibit compression fracture points within the experimental force range, indicating OUSMNs good toughness. Considering the viscosity of the matrix solution, mechanical properties, and UCL intensity, we optimized the precursor concentration of OUSMNs in water to 5 mg/mL for optimal performance.

The OUSMNs demonstrated excellent insertion capability into freshly excised keloids (within 4 h post-surgery). Upon removal, the keloid surface exhibited a complete 10×10 microneedle array with a 100% puncture rate (**Figure 2H**). Histological analysis (**Figure 2I**) confirmed that the OUSMNs penetrated the keloid to a depth of approximately 170 µm, effectively breaking through the skin barrier of the stratum corneum and reaching the superficial dermis. These exceptional mechanical properties positioned OUSMNs as a promising strategy for effective transdermal delivery of targeting keloids, highlighting considerable clinical application prospects.

In addition, the presence of OUSMNs did not compromise the viability of KFs (**Figure S19**), accompanied by strong biocompatibility. Furthermore, skin repair experiments *in vivo* also confirmed that OUSMNs application was minimally invasive and suitable for frequent use (**Figure S20**). Altogether, OUSMNs possessed the capabilities of excellent mechanical performance and biosafety, exhibiting promising applications of transdermal drug delivery in keloid.

Excised human skin remains the gold standard for *ex vivo* drug permeation studies 38. For this purpose, we evaluated the diffusion characteristics of OUSMNs, with and without the HKN_15_ formulation, in keloid tissues (**Figure 2J**). UCL images revealed that 4 h post-intervention, OUSMNs loaded with HKN_15_ diffused to a depth of approximately 500 μm, dramatically surpassing the 300 μm diffusion depth observed with OUSMNs without HKN_15_ (**Figure 2K**). These results emphasized the capability of OUSMNs to penetrate dense keloid tissues, and to deliver UCNPs into the dermis, with HKN_15_-modified formulations achieving substantially greater diffusion.

### Endogenous Ferroptosis Induced by OUSMNs

**Figure 3:**
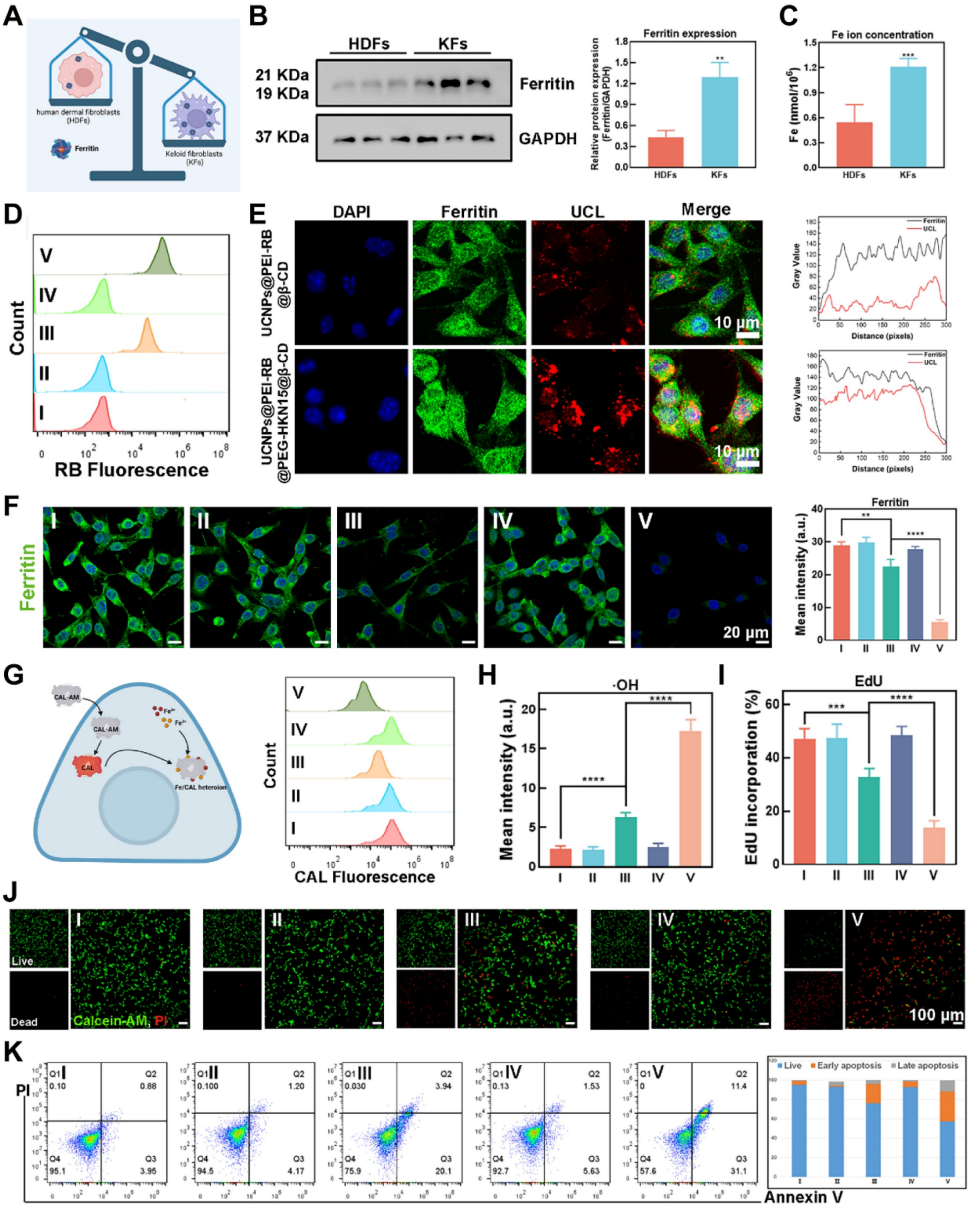
Endogenous ferroptosis induced by OUSMNs. (A) Illustration of ferritin expression differences between human dermal cells (HDFs) and KFs. (B) Western blot and densitometric analysis showed ferritin levels in HDFs and KFs. (C) Comparison of iron ion content between HDFs and KFs. (D) FCM analysis of cellular uptake after 4 h incubation. (E) Analysis of the intracellular co-localization of ferritin and OUSMNs. (F) CLSM images of Ferritin-stained KFs treated with groups Ⅰ-Ⅴ, and the corresponding quantitative analysis. (G) Schematic diagram and FCM analysis of intracellular iron ion content detection. (H) Quantitative analysis of ·OH generation determined by CLSM. (I) Quantitative analysis of cell proliferation using EdU as an indicator, measured by CLSM. (J) CLSM images of KFs after treatments with groups Ⅰ-Ⅴ. Green fluorescence: Calcein-AM represented live cells; red fluorescence: PI represented dead cells. (K) Annexin V-FITC/PI apoptosis assay and the corresponding quantitative analysis of apoptosis rate measured by FCM. PBS (Group I), SCD (Group II), UCNPs@PEI-RB&SCD (Group III), UCNPs@PEG-HKN_15_&SCD (Group IV), or UCNPs@PEI-RB@PEG-HKN_15_&SCD (Group V, OUSMNs). Mean ± SD, n = 3. **P < 0.01, ***P < 0.001, ****P < 0.0001.

Gene expression of ferritin, consisting of a protein shell surrounding an iron core containing 4500-5000 irons, was significantly upregulated in keloid tissues compared to normal skin tissues through bioinformatics analysis (**Figure 3A and Figure S21**). As demonstrated by immunofluorescence (IF) analysis (**Figure S22**) and western blotting (WB) assay (**Figure 3B-C**), KFs were reservoirs for triggering endogenous ferroptosis without needing exogenous irons. Hence, the crucial issue of inducing endogenous ferroptosis was the disruption of ferritin in KFs, which could be settled by applying OUSMNs.

First, as outlined earlier, the OUSMNs with HKN_15_-modified formulations effectively penetrate dense keloid tissue, delivering surface-functionalized UCNPs to the dermis and achieving broader diffusion. To explore the most efficient method for disrupting ferritin in KFs, we conducted interventions across five experimental groups. Among these, the group V (OUSMNs) was the most crucial, due to the combined targeting effect of HKN_15_ and the photosensitive properties of RB. We monitored the time-dependent uptake of surface-functionalized UCNPs in KFs using a two-photon confocal laser scanning microscope (CLSM). After endocytosis, the surface-functionalized UCNPs primarily localized to the cytoplasm. Additionally, the intracellular UCL fluorescence intensity increased with longer incubation, reaching a peak at 4 hours (**Figure S23**). Flow cytometry (FCM) analysis also showed that KFs treated with OUSMNs exhibited the highest fluorescence emission (**Figure 3D**). Subsequently, the co-localization of ferritin with surface-functionalized UCNPs was observed by two-photon CLSM. There were distinct yellow signals resulting from red-color UCL extensively overlaps with green-color fluorescence of the ferritin in the HKN_15_-modified group, suggesting that the HKN_15_-modified UCNPs internalized by KFs were localized around ferritin (**Figure 3E, left**). The colocalization analysis obtained the same results (**Figure 3E, right**). These findings confirmed that HKN_15_-modified UCNPs could specifically recognize and accumulate around ferritin in KFs, essential for the subsequent induction of endogenous ferroptosis. Gradually, there was a notable difference in ferritin degradation between the HKN_15_-modified group and the other groups through CLSM images (**Figure 3F**), thus implying a preferable method of ferritin disruption in KFs with targeted the effect of HKN_15_. Subsequently, more accumulation of a large number of free irons in the cytoplasm was detected by using Calcein (CAL)** (Figure 3G)**. Furthermore, the intracellular ·OH levels, generated via the Fenton reaction, exhibited much higher in the HKN_15_-modified group by hydroxyphenyl fluorescein **(Figure 3H** and **Figure S24)**, hence demonstrating that the designed surface-functionalized UCNPs had great potential to induce endogenous ferroptosis under 808 nm laser irradiation.

In causality succession, the cell proliferation ratio of KFs in the HKN_15_-modified group was much lower than the other groups shown by the EdU probe **(Figure 3I** and **Figure S25)**. Similarly, there was a phenomenon that more dead KFs were in the HKN_15_ and RB-modified group than those in the UCNPs@PEI-RB&SCD group, while the presence of dead KFs in the other group was minimal by Calcein-AM/PI double staining method **(Figure 3J)**. It was indicated that RB was the crucial factor of PDT-induced cell death and HKN_15_ with targeting effect will develop synergistic effect. And, the highest distinct signals of early apoptosis (Q3 quadrant; 31.1%) and late apoptosis (Q2 quadrant; 11.4%) were in group V by FCM, confirming the pro-apoptotic potential of endogenous ferroptosis and synergistic PDT (**Figure 3K**). The above results all demonstrated that OUSMNs showed powerful endogenous ferroptosis induction and synergistic PDT in KFs.

### Therapy Efficacy of OUSMNs-induced Endogenous Ferroptosis in KFs

**Figure 4:**
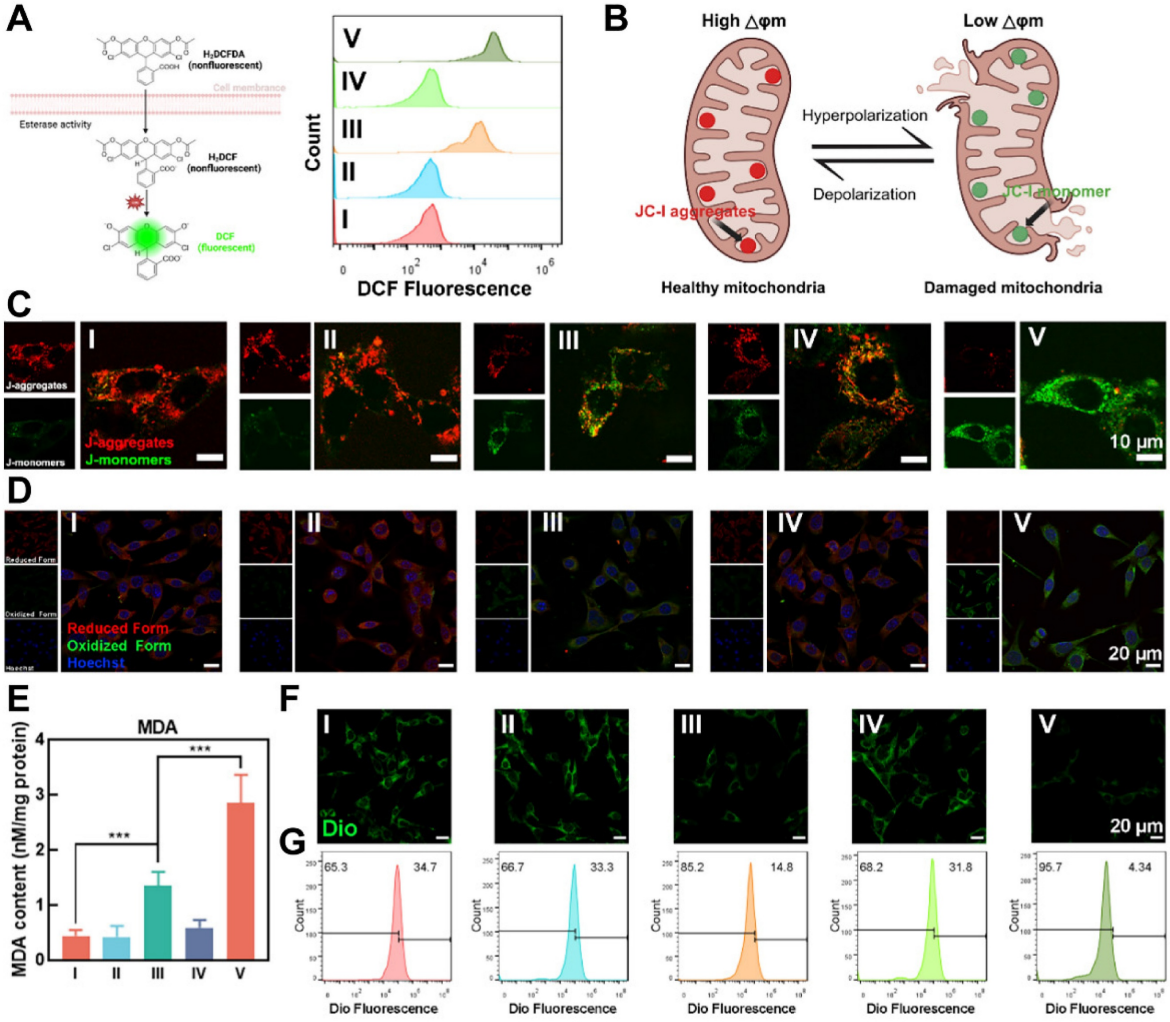
Therapy efficacy of OUSMNs-induced endogenous ferroptosis in KFs. (A) Schematic diagram and FCM analysis of KFs staining with DCFH-DA probe. (B) Diagram illustrating membrane potential changes during mitochondrial damage. (C) CLSM images of JC-1 stained KFs in groups Ⅰ-Ⅴ. Red fluorescence: JC-1 aggregates in healthy mitochondria. Green fluorescence: JC-1 monomers in damaged mitochondria. (D) CLSM images of C11-BODIPY^581/591^ stained KFs in groups Ⅰ-Ⅴ. Increased green fluorescence and decreased red fluorescence indicated LPO production. (E) Quantitative analysis of MDA content in KFs treated with groups Ⅰ-Ⅴ. (F) CLSM images and (G) FCM analysis of KFs staining with Dio probe. Decreased green fluorescence intensity indicated cell membrane damage. Mean ± SD, n = 3. **P < 0.01, ***P < 0.001, ****P < 0.0001.

The effective destruction of ferritin by reactive oxygen species is the key to induce endogenous ferroptosis 39. The non-polar probe DCFH-DA could be used to readily penetrate cell membranes, and then hydrolyzed by endoplasmic reticulum esterases into dichlorodihydrofluorescein (DCFH) in KFs. DCFH was then oxidized by intracellular ROS into highly fluorescent dichlorofluorescein (DCF), emitting a strong signal. We observed that Group V produced a significantly higher amount of ROS compared to Groups I-IV, which could be attributed to the activation of endogenous ferroptosis, triggering the Fenton reaction** (Figure 4A)**.

Mitochondrial membrane potential is a critical indicator of mitochondrial function and cellular health 40. As illustrated in **Figure 4B**, red fluorescence indicates JC-1 aggregates in healthy mitochondria with natural membrane potential, whereas green fluorescence indicates JC-1 monomers in damaged mitochondria with reduced membrane potential. **Figure 4C** showed that in groups I, II, and IV, the presence of bright red fluorescence (JC-1 aggregates) and weak green fluorescence (JC-1 monomers) portended natural mitochondrial membrane potential and healthy KFs. In contrast, OUSMNs exhibited a much stronger green fluorescence and nearly absent red fluorescence, indicating severe mitochondrial dysfunction caused by endogenous ferroptosis and synergies PDT.

LPO and MDA, the final product of lipid oxidation, are key biomarkers of ferroptosis 41. To detect LPO levels, we used BODIPY^581/591^-C11, a fluorescent probe that exhibits red fluorescence in its reduced form and green fluorescence when oxidized, indicating the presence of LPO 42. Thus, by tracking the color changes of BODIPY^581/591^-C11, the intracellular levels of LPO can be indirectly quantified, providing a method for assessing oxidative stress and cellular damage. Group V exhibited bright green fluorescence and nearly negligible red fluorescence, indicating a high level of LPO in KFs** (Figure 4D)**. In contrast, little to no fluorescence change was observed in Groups I, II, and IV. This trend was also evident in the MDA assay (**Figure 4E**). These findings confirmed that the OUSMNs effectively induced LPO, likely due to the synergistic effects of the endogenous ferroptosis-mediated Fenton reaction and NIR light-triggered synergistic PDT, which together enhanced ROS production.

The impact of LPO on KFs membrane integrity were clearly demonstrated using DiO probe. **Figure 4F** presented CLSM images of KFs stained with a DiO probe, which emitted strong green fluorescence when incorporated into the intact phospholipid bilayer of the cell membrane. Membrane damage was most pronounced in Group IV, which exhibited significant disruption compared to other groups, attributed to LPO generation. To further quantify membrane integrity, we also used FCM to measure DiO fluorescence intensity in KFs after treatment. As shown in **Figure 4G**, OUSMNs exhibited significantly lower fluorescence intensity compared to Groups I-IV, aligning with the CLSM results. These findings confirmed that LPO generation, driven by endogenous ferroptosis and synergistic PDT, played a critical role in disrupting cell membrane integrity. These results confirmed that OUSMNs were capable of spatiotemporal destruction of ferritin and NIR light-triggered synergistic PDT in KFs.

**Figure 5:**
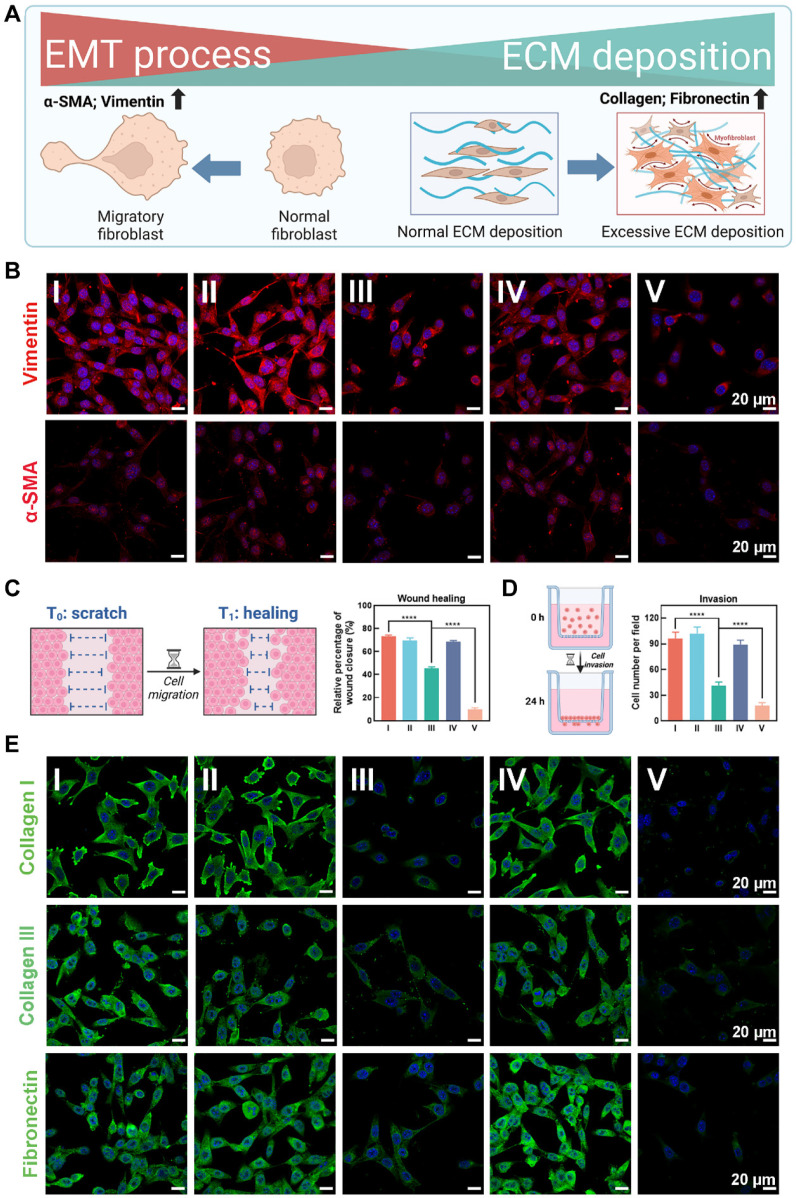
Inhibiting ECM deposition and EMT processes of KFs. (A) Diagram illustrating ECM deposition and EMT processes in the pathological progression of keloids. (B) CLSM images of EMT-related markers α-SMA and vimentin stained KFs in groups Ⅰ-Ⅴ. (C) Diagram illustrating cell scratch experiments and corresponding quantitative analysis. (D) Diagram illustrating transwell invasion assay and corresponding quantitative analysis. (E) CLSM images of ECM-related markers (Collagen I, Collagen III, and Fibronectin) stained KFs in groups Ⅰ-Ⅴ. Mean ± SD, n = 3. **P < 0.01, ***P < 0.001, ****P < 0.0001.

The extracellular matrix (ECM) deposition and epithelial-to-mesenchymal transition (EMT) processes are crucial pathological phenotypes in the formation and progression of keloids 43. Excessive ECM deposition led to keloid hyperplasia, resulting in thickened, raised skin that severely affected both appearance and function 4. Additionally, EMT processes has been reported to drive fibroblast phenotypic modifications and stemness acquisition, contributing to invasiveness and recurrence in keloids 44. Therefore, targeting and reversing ECM deposition and EMT processes are critical strategies for inhibiting keloid hyperplasia and preventing recurrence. As illustrated in **Figure 5A**, keloid tissue exhibited marked overexpression of EMT markers α-SMA and vimentin, and ECM markers collagen I, collagen III, and fibronectin compared to normal skin, reflecting the pathological features of keloid hyperplasia and invasiveness.

The CLSM images showed that the EMT-related markers α-SMA and vimentin were most significant decreased after treatment with OUSMNs in KFs, compared with other treatment groups (I-IV) due to the endogenous ferroptosis and synergistic PDT** (Figure 5B)**. Considering the continuous stages involving cell proliferation and migration in the formation of keloids 45, we further explored the potential role of OUSMNs in wound healing *in vitro*. The findings highlighted that endogenous ferroptosis significantly impeded KF wound closure (**Figure 5C and Figure S26**), the same as observed in the transwell invasion assay (**Figure 5D and Figure S27**).

**Figure 5E** presented CLSM images of ECM-related markers collagen I, collagen III, and fibronectin-stained KFs treated with groups Ⅰ-Ⅴ. The decrease in green fluorescence revealed the inhibition of ECM deposition. The differences between group V and I-IV were significant, ascribed to the integration of endogenous ferroptosis with NIR light-triggered synergistic PDT.

The above findings showed that triggering endogenous ferroptosis and synergistic PDT could inhibit critical pathogenic phenotypes in KFs, such as ECM deposition and the EMT process, for mitigating keloid hyperplasia and invasiveness.

### Mechanism of OUSMNs-induced Endogenous Ferroptosis

To further investigate the molecular biological mechanisms of OUSMNs, we conducted a transcriptomic analysis to examine mRNA expression changes in KFs after the corresponding treatments. The volcano plot illustrated the distinct transcriptomic discrepancy between the NIR-triggered OUSMNs group and control groups (**Figure S28**), revealing a total of 1527 differentially expressed genes (DEGs), comprising 671 upregulated mRNAs and 856 downregulated mRNAs. The several pivotal DEGs were involved in cell apoptosis, ferroptosis, ECM deposition, and EMT process were notably identified (**Figure S29-32**).

**Figure 6:**
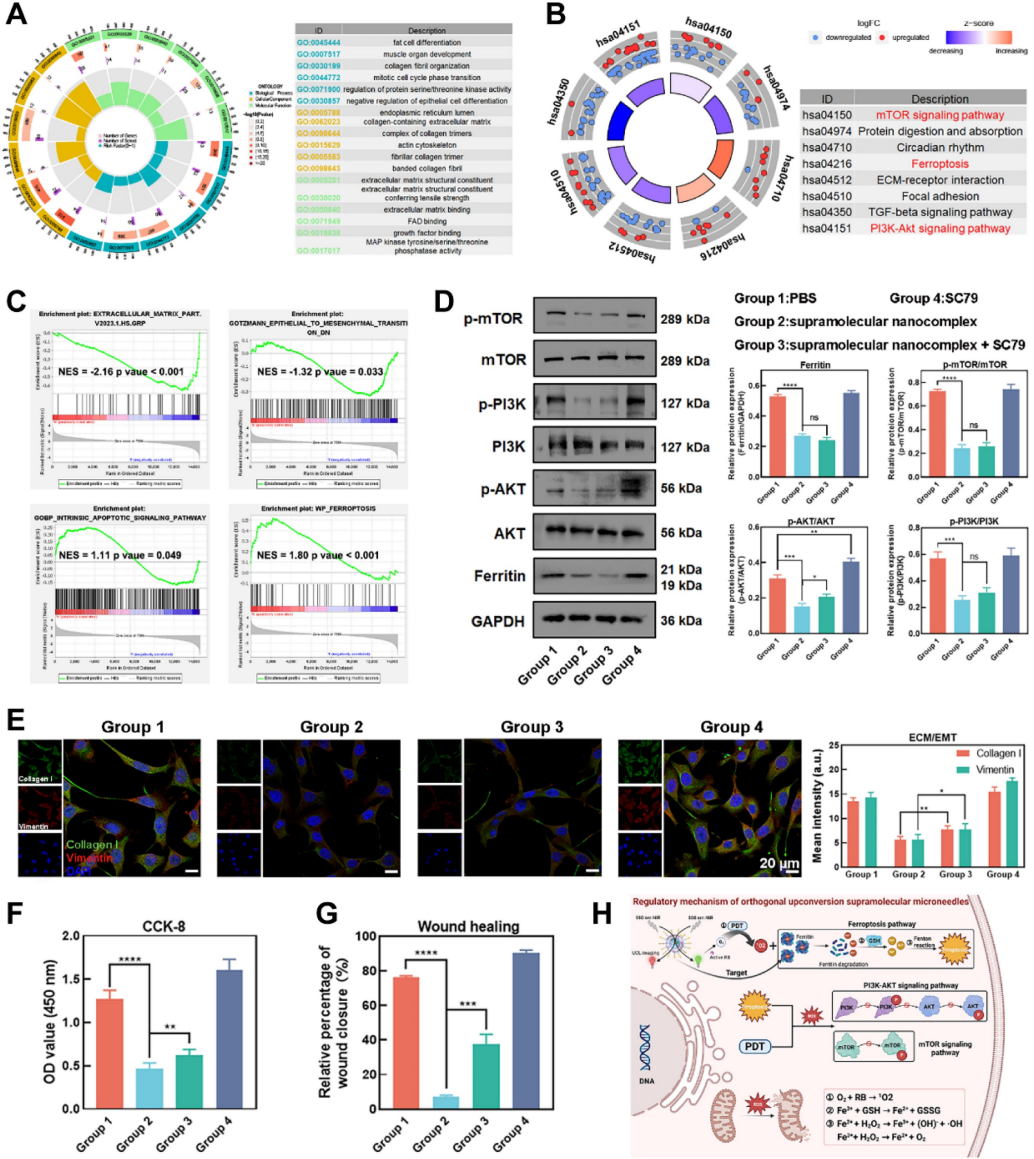
Mechanism of OUSMNs-induced endogenous ferroptosis. (A) GO analysis and (B) KEGG analysis of DEGs. (C) Enrichment plots from GSEA analysis of genesets for ECM deposition, EMT processes, apoptosis, and ferroptosis. NES, normalized enrichment score. |NES| > 1 and p value < 0.05 were considered as significant difference in the two groups. (D) Western blot analysis was conducted to measure p-AKT, p-PI3K, p-mTOR, and ferritin expression protein levels. (E) IF staining (F) CCK-8 assay, and (G) transwell assay were performed as part of the rescue experiments using SC79. (H) Regulatory mechanism of OUSMNs. PBS (group 1), UCNPs@PEI-RB@PEG-HKN_15_&SCD + 808 nm NIR (group 2), UCNPs@PEI-RB@PEG-HKN_15_&SCD + 808 nm NIR + SC79 (group 3), or SC79 (group 4). Mean ± SD, n = 3. **P < 0.01, ***P < 0.001, ****P < 0.0001.

Next, Gene Ontology (GO) and Kyoto Encyclopedia of Genes and Genomes (KEGG) analyses were conducted to identify the biological functions and potential regulatory pathways enriched by the DEGs 46. The GO analysis revealed that the DEGs were enriched in biological functions related to wound healing, ECM deposition, and cell proliferation, indicating the critical role of OUSMNs in regulating pathological phenotype (**Figure 6A**). Furthermore, KEGG analysis further provided novel insights into the potential regulatory mechanisms and intracellular impact of the OUSMNs-induced therapeutic effects in keloids. The DEGs were enriched in the mTOR signaling pathway, the ferroptosis signaling pathway, and the PI3K-AKT signaling pathway (**Figure 6B**). Considering the expression patterns of entire gene sets, gene set enrichment analysis (GSEA) further confirmed that the OUSMNs could significantly impact the ECM deposition, EMT processes, apoptosis, and ferroptosis in KFs (**Figure 6C**).

Based on the KEGG and GSEA enrichment analyses, we sought to provide in-depth and reliable evidence for OUSMNs-induced mechanism in keloid therapy. The WB results revealed that the relative expression levels of p-AKT, p-PI3K, p-mTOR, and ferritin decreased after OUSMNs treatment in KFs **(Figure 6D)**. The signal pathway rescue experiment was considered the gold standard for assessing the pathway criticality in pathological processes. Thus, we performed rescue experiments using the AKT activator (SC79, 10 μM). WB analysis showed that SC79 could partially recover the inhibitory effect of OUSMNs on p-AKT, while did not affect p-mTOR and ferritin levels **(Figure 6D)**.

Moreover, we further investigated the effects of PI3K-AKT pathway, mTOR pathway, and ferroptosis pathway on cell proliferation and migration, ECM deposition, and EMT processes. OUSMNs reduced the expression levels of ECM-related Collagen I and EMT-related vimentin, and this effect could be reversed by SC79-medicated p-AKT activation (**Figure 6E**). The CCK-8 and wound healing assays also revealed that SC79 could partly restore cell viability and migration capacity after OUSMNs treatment (**Figure 6F-G**). Here, we confirmed that OUSMNs could inhibit KF functions mediated by inhibiting the PI3K-AKT and mTOR pathways while activating the ferroptosis pathway (**Figure 6H**).

### Therapy Efficacy of OUSMNs in Xenograft Keloid Model in Nude Mice

**Figure 7:**
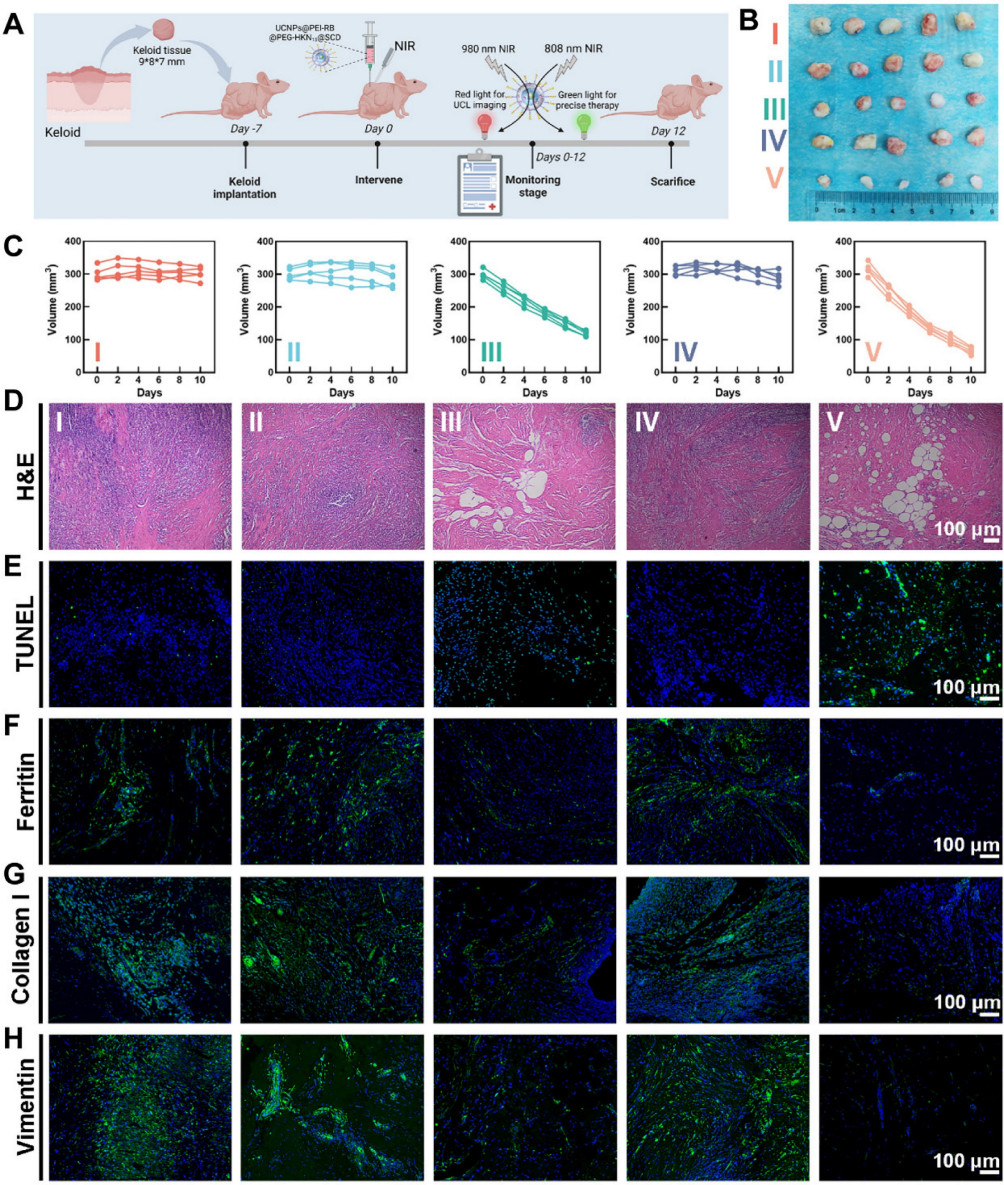
Therapy efficacy of OUSMNs in xenograft keloid model in nude mice. (A) Schematic diagram of the construction and intervention protocol in a keloid transplantation model in BALB/c nude mice. (B) Images showing keloid transplantations excised from the subcutaneous of nude mice at various treatment endpoints. (C) The growth curves of keloid transplantations in groups Ⅰ-Ⅴ. Histological observation of the keloid transplantations with staining of (D) H&E, (E) TUNEL, (F) Ferritin, (G) Collagen I, and (H) Vimentin after above-mentioned treatments (group I-V). PBS (Group I), SCD (Group II), UCNPs@PEI-RB&SCD (Group III), UCNPs@PEG-HKN_15_&SCD (Group IV), or UCNPs@PEI-RB@PEG-HKN_15_&SCD (Group V). Mean ± SD, n = 3. **P < 0.01, ***P < 0.001, ****P < 0.0001.

The therapy efficacy of various treatments was assessed using a keloid implantation model in BALB/c nude mice 47, as depicted in **Figure 7A**, which outlined the schedule of keloid transplantation and intervention strategies. Notably, the experimental design also included a positive control group that received only 808 nm NIR irradiation to exclude possible interference effects of NIR light itself. Results of weight and volume measurements, hematoxylin and eosin (H&E) staining, and IF staining confirmed that 808 nm infrared irradiation did not impair the viability of keloid grafts (**Figure S33**). **Figure 7B** presented photographs of keloid transplantations excised on the 12th-day post-treatment, and **Figure 7C** showed the growth curves of keloid implantations after different treatments. Compared with the other groups, the OUSMNs showed much more efficacious keloid inhibition (approximately 80% in volume and 75% in weight) due to the endogenous ferroptosis and PDT synergistically enhancing ROS production in keloids (**Figure S34**).

During the administration period, the body weight of nude mice in all treatment groups showed no significant abnormal fluctuations (**Figure S35**). The H&E staining of the major organs (heart, liver, spleen, lung, and kidney) to evaluate potential organ toxicity, showed negligible inflammatory lesions and pathological changes, thus confirming the excellent tissue compatibility and biosafety of the nanocomplex (**Figure S36**). Subsequently, by measuring the changes in white blood cell, red blood cell, and platelet counts in the whole blood of nude mice across various intervention groups, we confirmed that the OUSMNs showed little impact on the hematopoietic function and immune status of the mice** (Figure S37)**.

**Figure 7D-F** presented the histological analysis of keloid transplantations in groups I-V following H&E, TUNEL, or ferritin staining. Notably, compared to other treatment groups, the OUSMNs group demonstrated more severe cell apoptosis and necrosis in H&E and TUNEL staining images and more obvious ferritin destruction in ferritin staining image.

Furthermore, collagen I and vimentin were stained using IF to acquire a comprehensive knowledge of the internal mechanism of keloid hyperplasia and invasive pathological phenotype. For keloid implantations treated with OUSMNs, there was the most significant reduction in collagen I (**Figure 7G**) and vimentin (**Figure 7H**) expression, demonstrating that endogenous ferroptosis and synergistic PDT could markedly inhibit the pathological phenotypes of ECM and EMT *in vivo*. The results of western blot also demonstrated that the combination of endogenous ferroptosis with PDT induced the most significant collagen degradation** (Figure S38)**. Therefore, these *in vivo* results collectively suggested that the OUSMNs effectively inhibited the pathological phenotype of keloid implantations through triggering endogenous ferroptosis and synergistic PDT, for superior therapeutic efficacy in keloids.

Building on these findings, our study delineates the therapeutic potential of OUSMNs in modulating endogenous ferroptosis for keloid regression, yet several limitations warrant cautious interpretation. A notable constraint is the absence of definitive validation using ferroptosis-specific inhibitors (e.g., ferrostatin-1 48, liproxstatin-1 49), which are indispensable for identifying ferroptosis from multiple programmed cell death pathways (such as apoptosis and necrosis) and establishing mechanistic causality between ROS accumulation, ferritin degradation, and therapeutic efficacy 50. Nevertheless, the synchronized surge in lipid peroxidation markers (including MDA and BODIPY staining) alongside ferritin depletion provides pathophysiological coherence to our hypothesis of endogenous ferroptosis-driven keloid resolution. Despite the lack of direct evidence of pharmacologic inhibition, this synergistic change in the marker network has suggested from a pathophysiologic level that endogenous ferroptosis plays a central role in keloid therapy.

While the current most idealized experimental models—humanized systems co-engrafted with autologous immune cells and patient-derived xenografts 51—are recognized as the most physiologically relevant platforms for studying keloids, technical complexities (e.g., limited access to patient-matched immune cells, specialized infrastructure requirements) and resource constraints necessitated the use of a nude mouse xenograft model in this preliminary investigation. Despite its inherent limitations in recapitulating human immune-inflammatory crosstalk 52, this model successfully achieved our primary objective: validating the proof-of-concept efficacy of OUSMNs in triggering endogenous ferroptosis-dependent keloid regression. The robust biomarker concordance (lipid peroxidation, ferritin degradation) and therapeutic outcomes observed in this system provide a critical foundation for subsequent mechanistic studies in advanced humanized models. Future work will prioritize humanized systems models to fully elucidate the translational potential of this strategy.

Furthermore, despite OUSMNs' merits in targeted delivery and rapid dissolution, clinical scalability demands rigorous optimization of UCNP batch consistency and long-term biocompatibility. Collectively, while these limitations highlight avenues for refinement, our study establishes a proof-of-concept framework for utilizing endogenous ferroptosis and synergistic PDT, paving the way for minimally invasive precision therapies against fibroproliferative disorders.

## Conclusion

In this study, we developed strong and tough, rapidly dissolving OUSMNs, which were capable of effectively penetrating keloid tissue, rapidly releasing surface-functionalized UCNPs, and enabling anti-keloid therapy via endogenous ferroptosis and synergistic PDT. Moreover, the release of surface-functionalized UCNPs spatiotemporally disrupted ferritin through NIR regulation, and consequently generated abundant ROS through Fenton reaction and synergistic PDT, thereby effectively inhibiting excessive proliferation and invasive growth of the KFs. Furthermore, OUSMNs presented a promising strategy for utilizing intracellular ferritin as the endogenous source of iron to induce ferroptosis and realize the synergistic efficacy in xenograft keloid model of nude mice. Collectively, our well-established and validated OUSMNs possess excellent keloid-inhibited therapeutic functions and good biocompatibility, hopefully contributing to endogenous ferroptosis-triggered nanoconstruction strategy of keloids.

## Supplementary Material

Supplementary figures and tables.
