# Supplementary material for: Orthogonal upconversion supramolecular microneedles promote endogenous ferroptosis in keloids
Source: Theranostics. 2025 May 8;15(13):6184–202. doi: 10.7150/thno.108289 (PMC12159835; doi:10.7150/thno.108289)
Supplement: Supplementary file 1 — Supplementary figures and tables. [file thnov15p6184s1.pdf]

## Supporting Figures

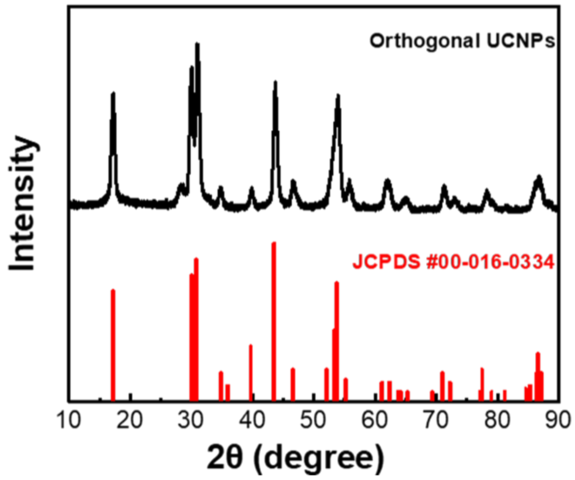


**Figure S1.** The XRD of orthogonal UCNPs.

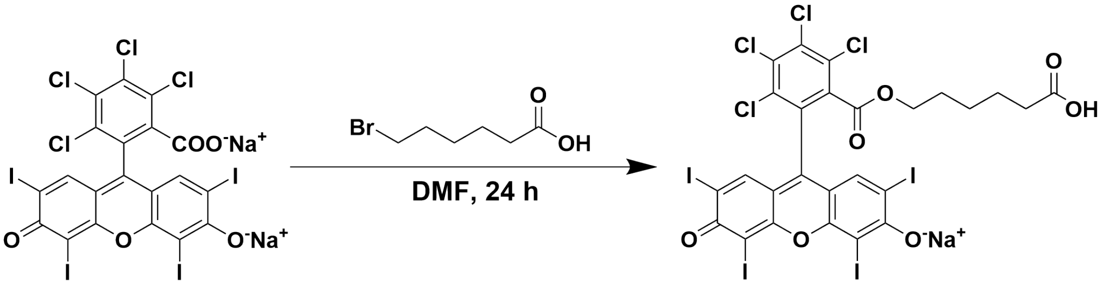


**Figure S2.** Synthetic scheme of RB-COOH.

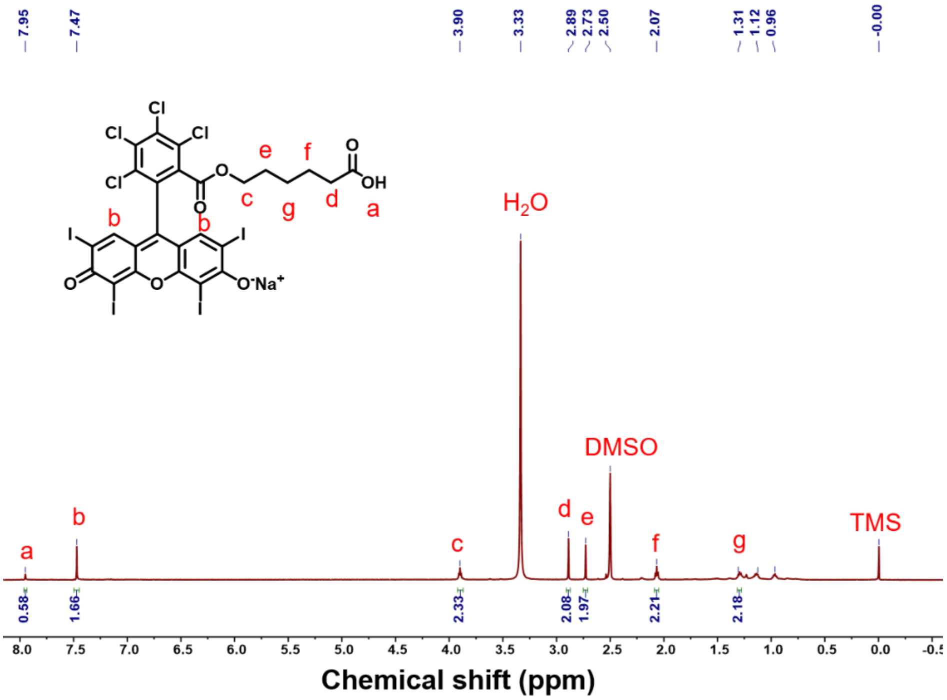


**Figure S3.** $^{1}$H NMR spectra of RB-COOH.

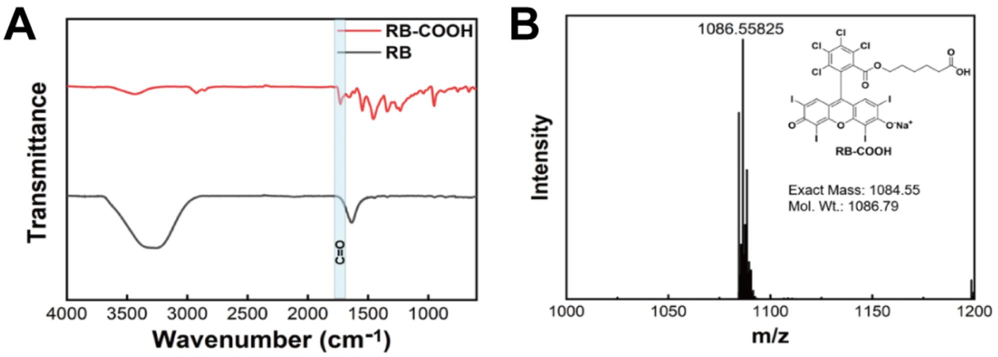


**Figure S4.** FT-IR (A) and HRMS mass spectrum (B) of RB-COOH.

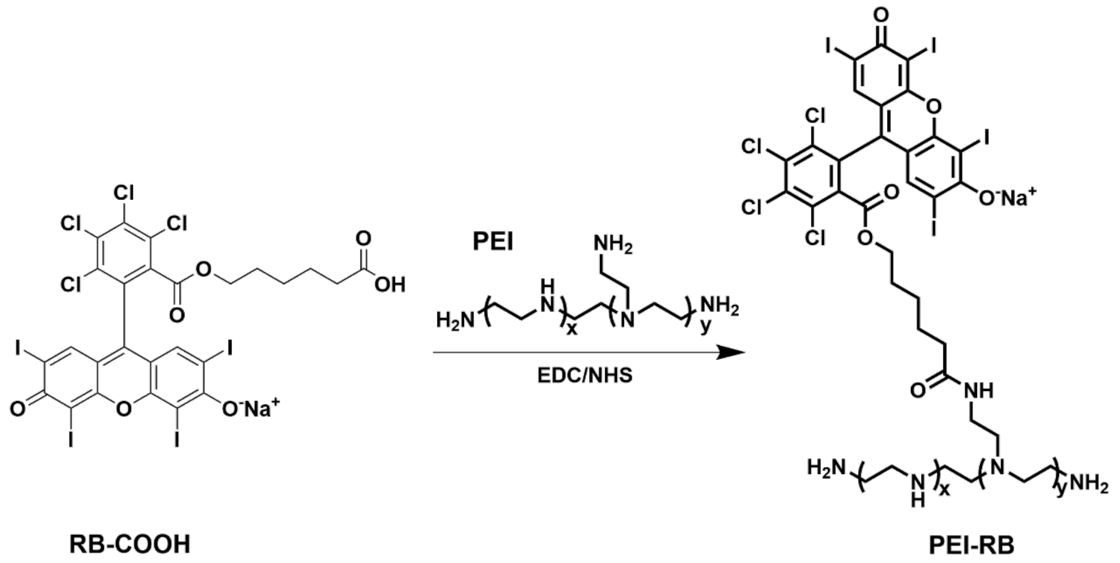


**Figure S5.** Synthetic scheme of PEI-RB.

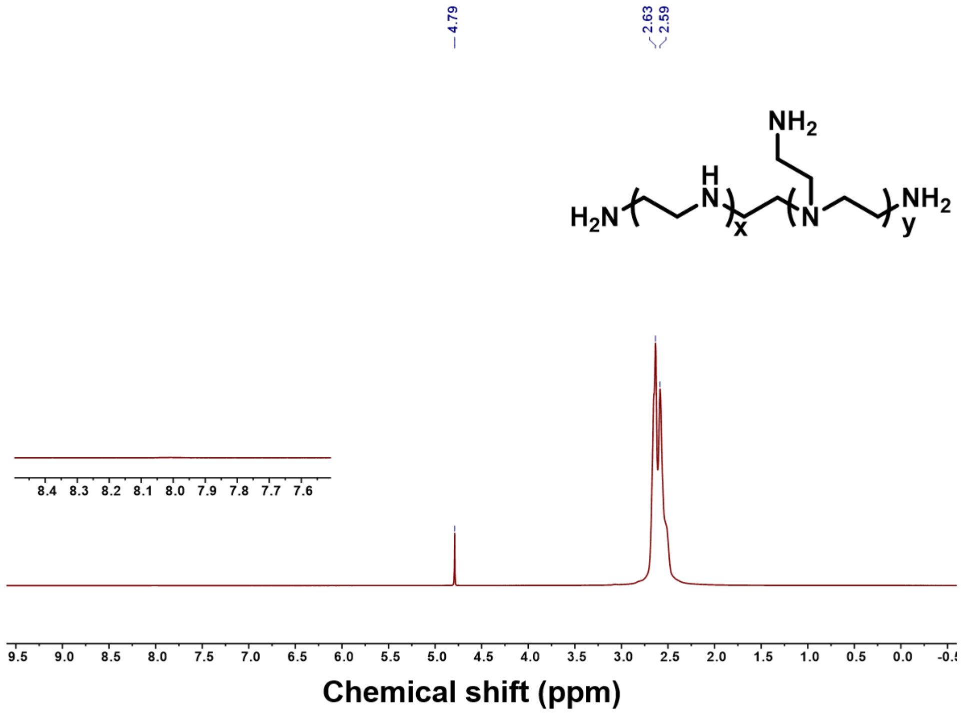


**Figure S6.** $^{1}$H NMR spectra of PEI.

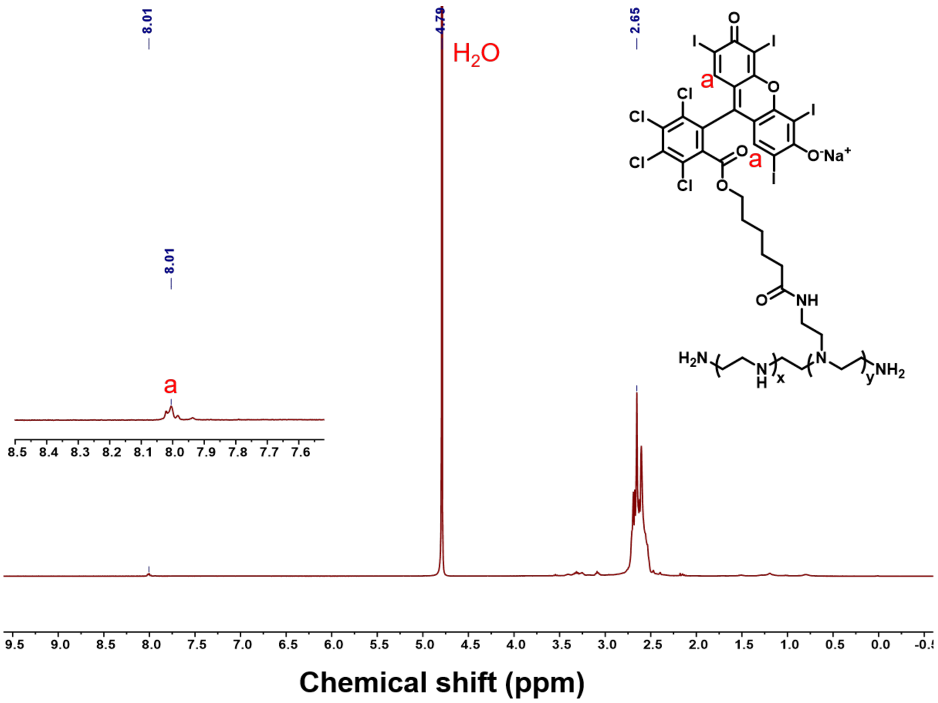


**Figure S7.** $^{1}$H NMR spectra of PEI-RB.

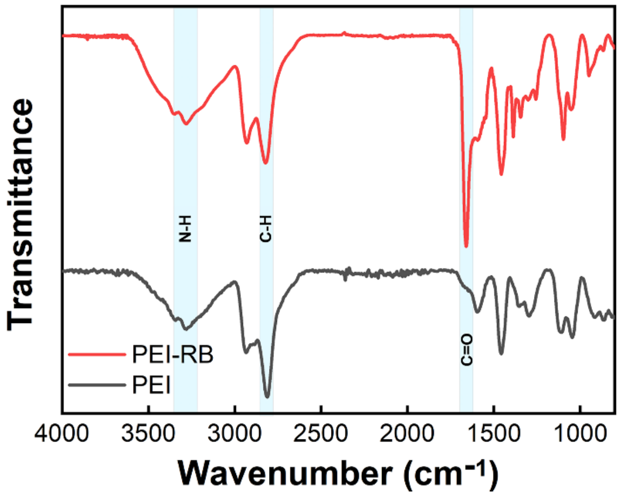


**Figure S8.** FT-IR of PEI and PEI-RB.

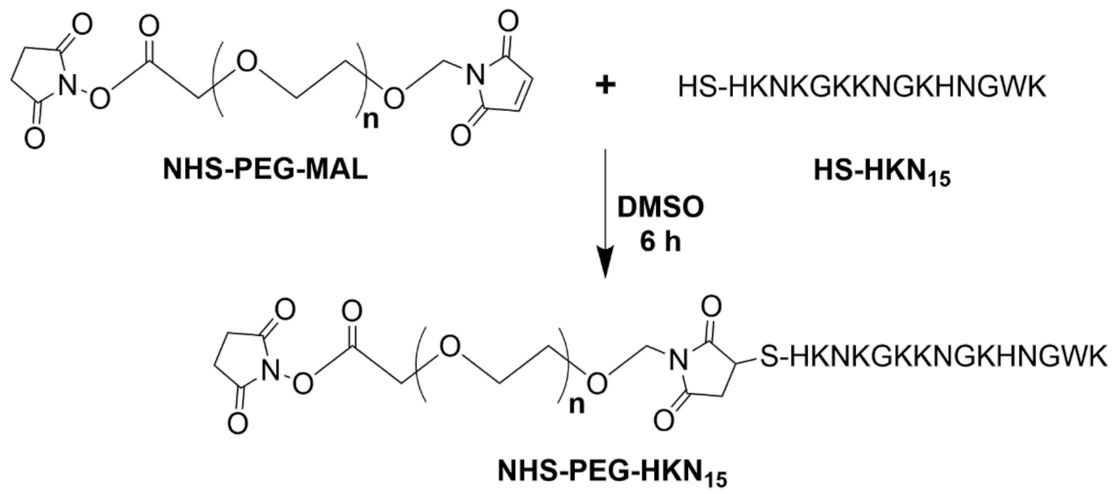


**Figure S9.** Synthetic scheme of NHS-PEG-$HKN_{15}$.

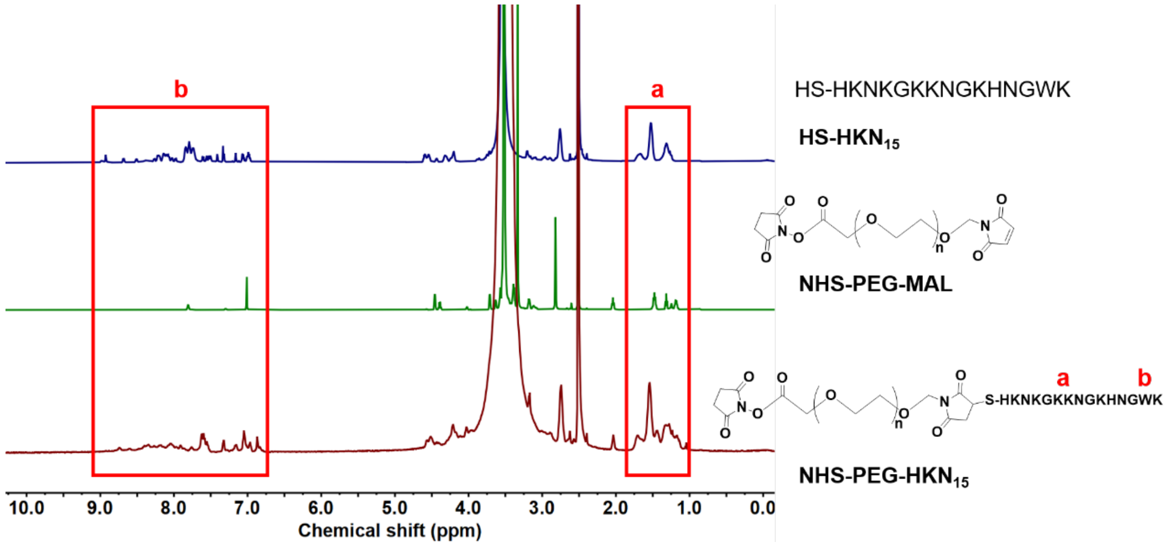


**Figure S10.** $^{1}$H NMR spectra of $HKN_{15}$, NHS-PEG-Mal, and NHS-PEG-$HKN_{15}$.

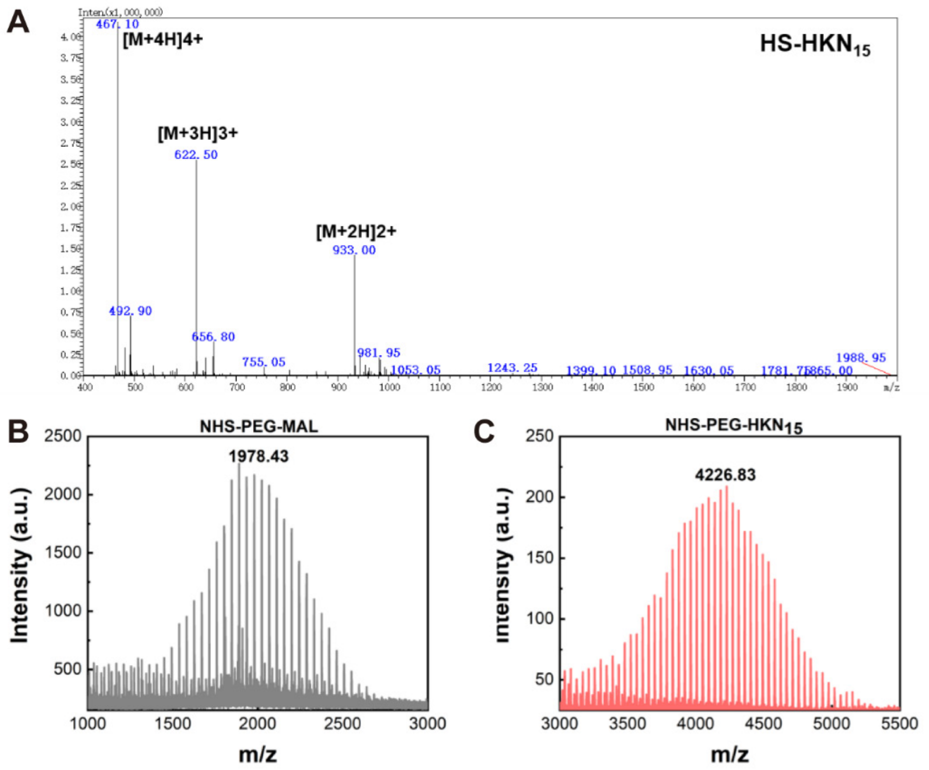


**Figure S11.** HRMS mass spectrum of (A) $HKN_{15}$, (B) NHS-PEG-Mal, and (C) NHS-PEG-$HKN_{15}$.

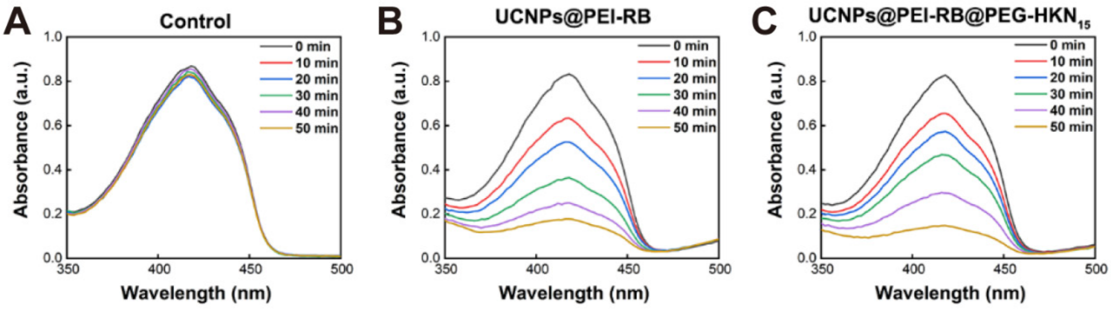


**Figure S12.** UV–vis assay of ROS generation using DPBF probe.

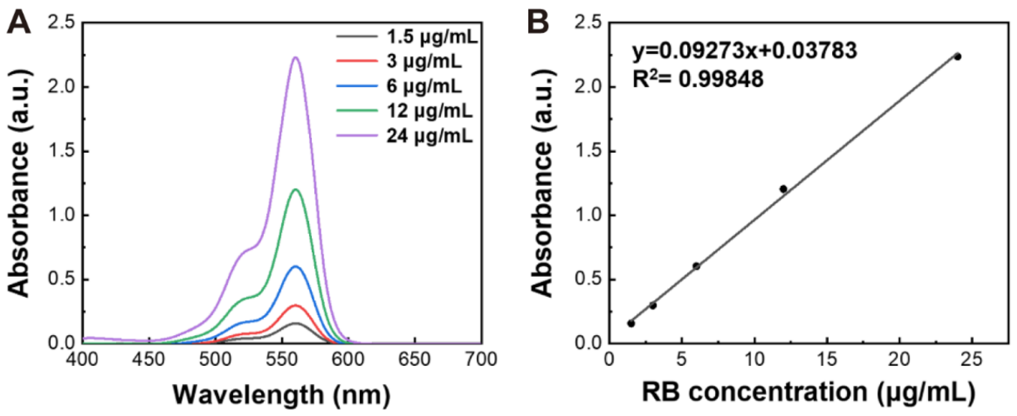


**Figure S13.** (A) UV-Vis absorption analysis and (B) the standard curve of RB

(absorbance at 560 nm).

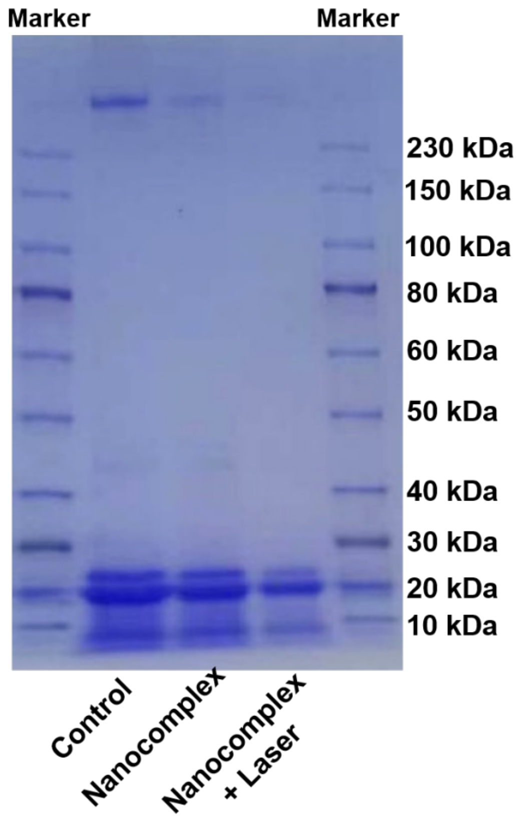


**Figure S14.** SDS-PAGE analysis of surface-functionalized UCNPs-induced ferritin degradation.

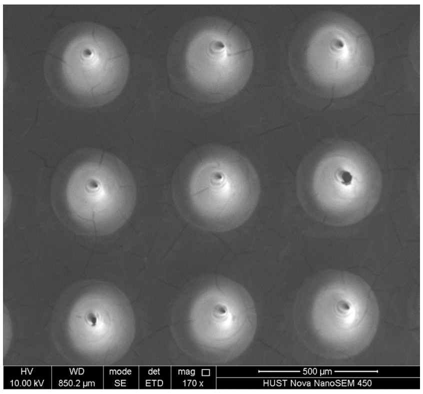


**Figure S15**. Scanning electron microscopy (SEM) images of OUSMNs.

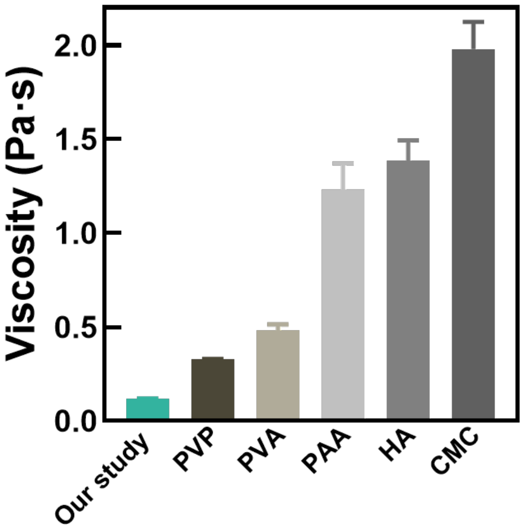


**Figure S16.** Comparison of matrix solution viscosity between OUSMNs and five commonly used polymer-based dissolving microneedles.

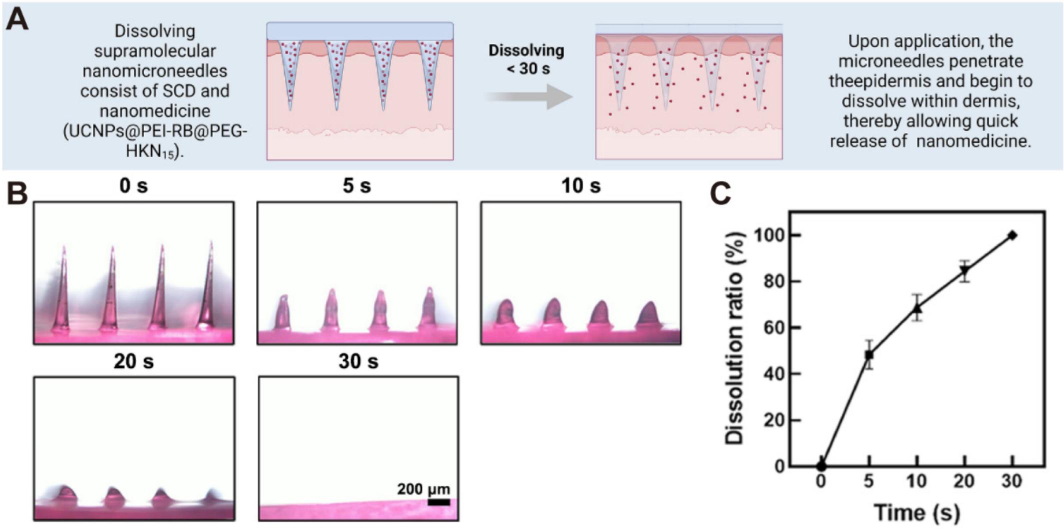


**Figure S17.** Dissolution rate of OUSMNs. (A) Diagram illustrating the dissolution mode of OUSMNs. (B) Dissolution of OUSMNs in a modified gelatin skin model over different durations. (C) Statistical chart of the dissolution rate of OUSMNs.

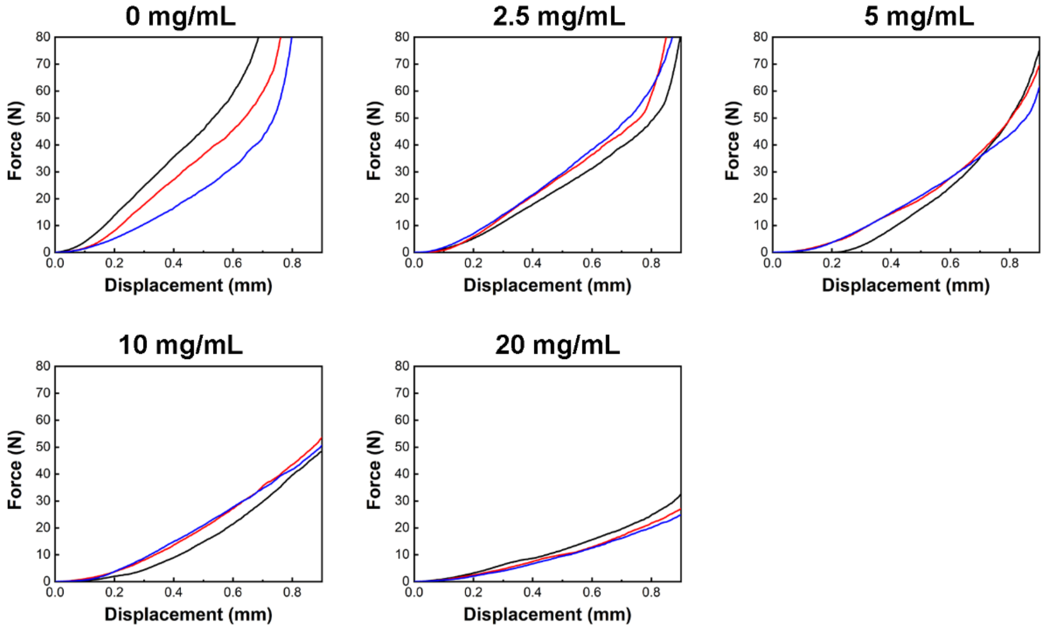


**Figure S18.** Comparison of compression force-displacement curves for various concentrations of OUSMNs.

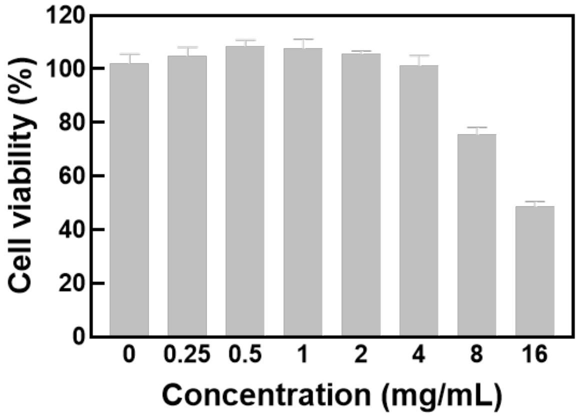


**Figure S19.** Biosafety evaluation of OUSMNs in KFs.

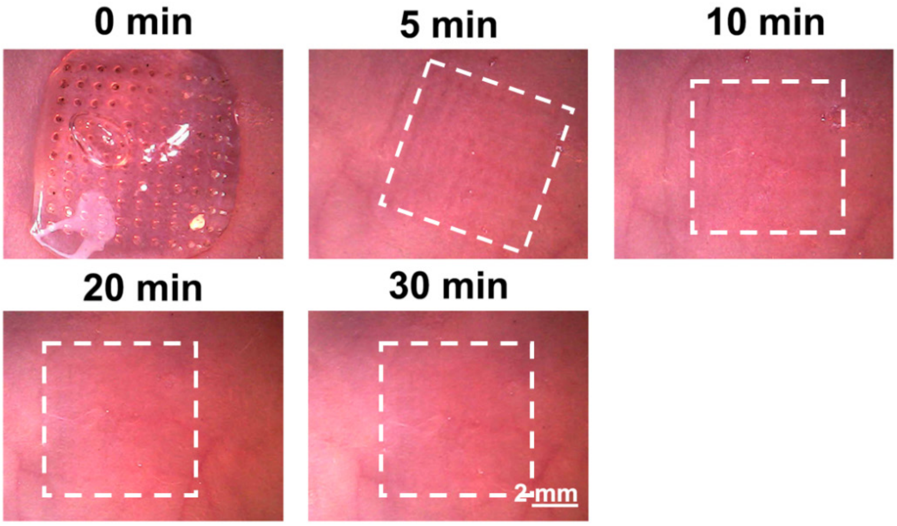


**Figure S20**. Skin recovery after application of OUSMNs.

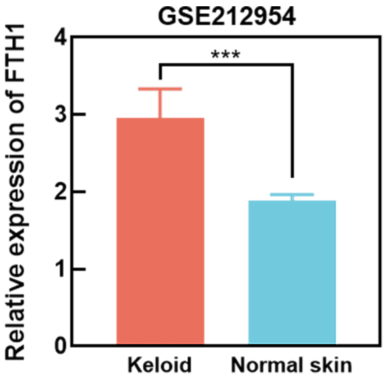


**Figure S21.** Bioinformatics analysis of ferritin (gene name: FTH1) gene expression levels in keloid tissues compared to normal skin tissues.

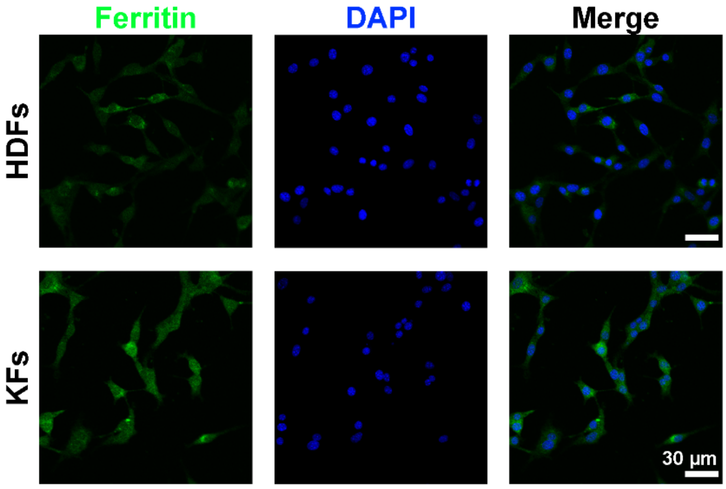


**Figure S22.** Immunofluorescence (IF) staining of ferritin in KFs and HDFs.

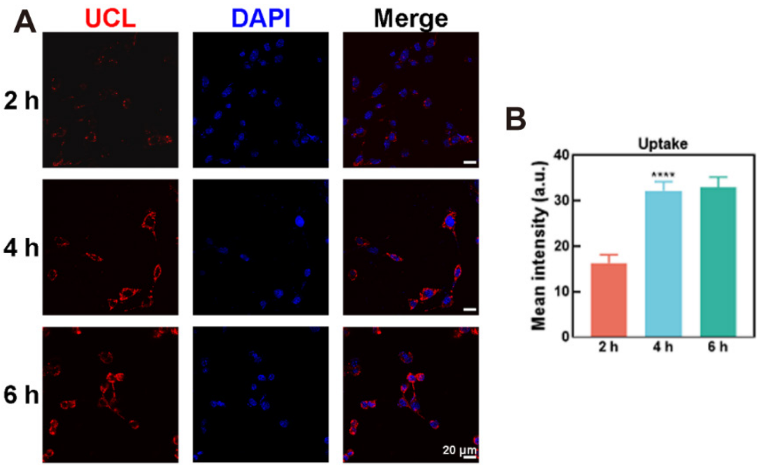


**Figure S23.** Evaluation of KFs uptake of OUSMNs using two-photon CLSM.

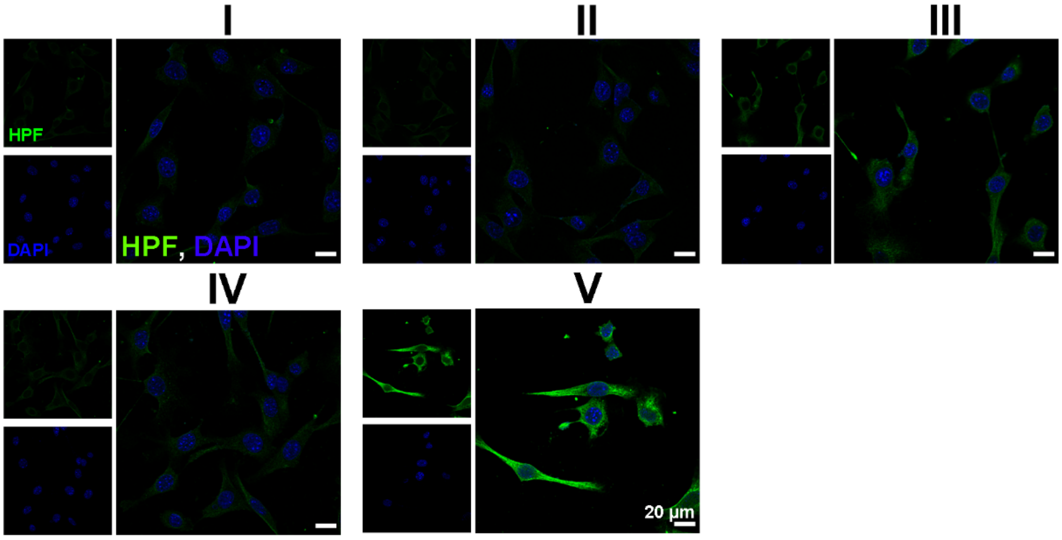


**Figure S24.** IF staining of HPF in KFs.

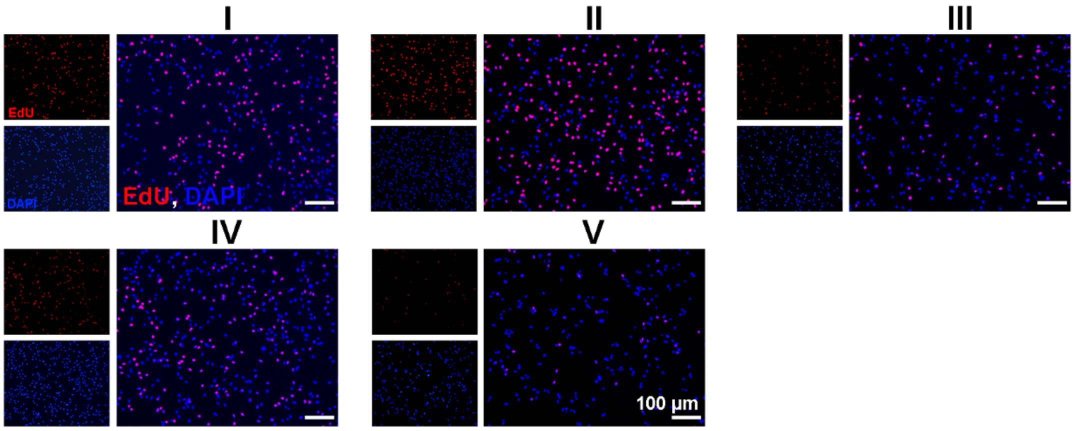


**Figure S25.** EdU staining was applied to assess cell proliferation, with EdU (red fluorescence) marking proliferating cells and DAPI (blue fluorescence) marking total

cells.

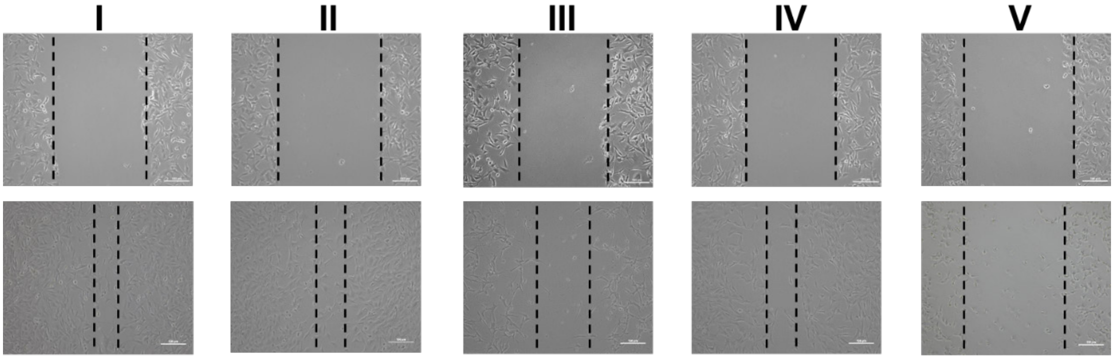


**Figure S26.** The cell scratch assay was used to evaluate cell migration capability. Scale bar=100 μm.

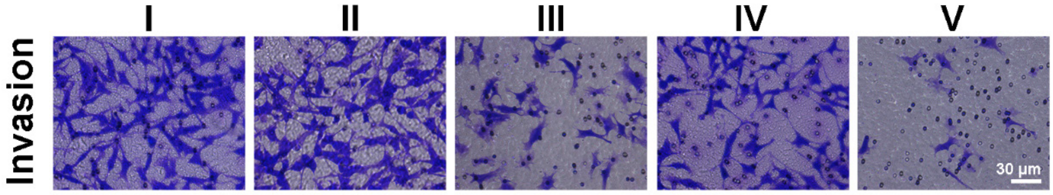


**Figure S27.** The Transwell invasion assay was used to evaluate cell invasive capacity.

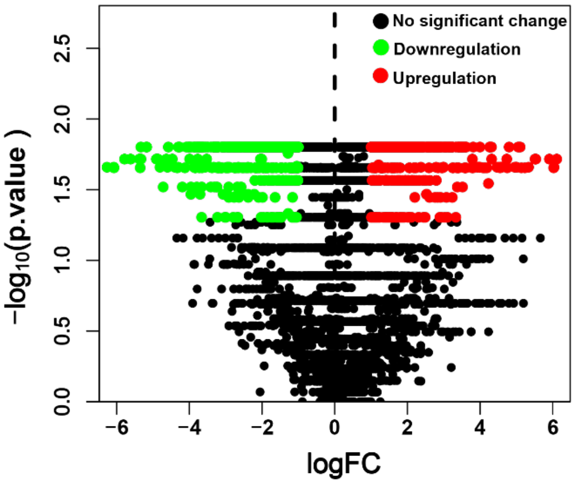


**Figure S28.** The volcano plot depicted differentially expressed genes.

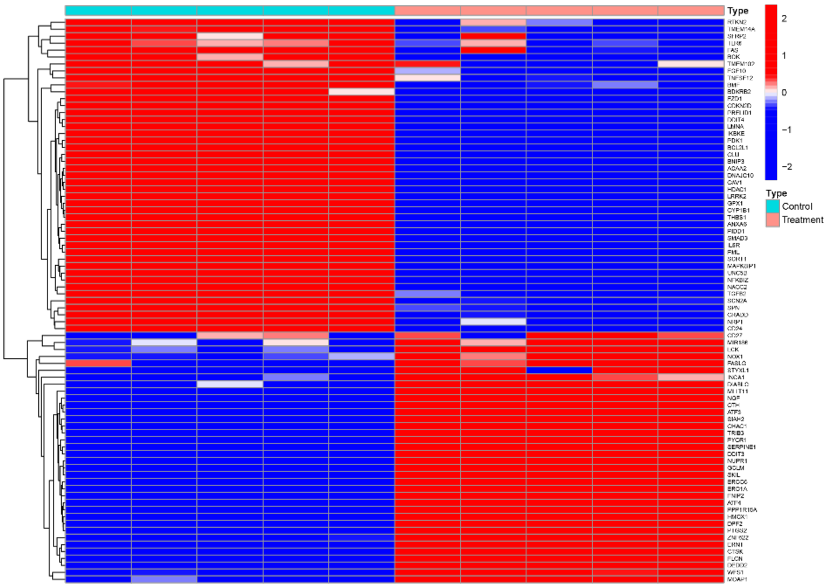


**Figure S29.** Heatmap of markers for cell apoptotic process selected from the mRNA sequencing data after OUSMNs treatment.

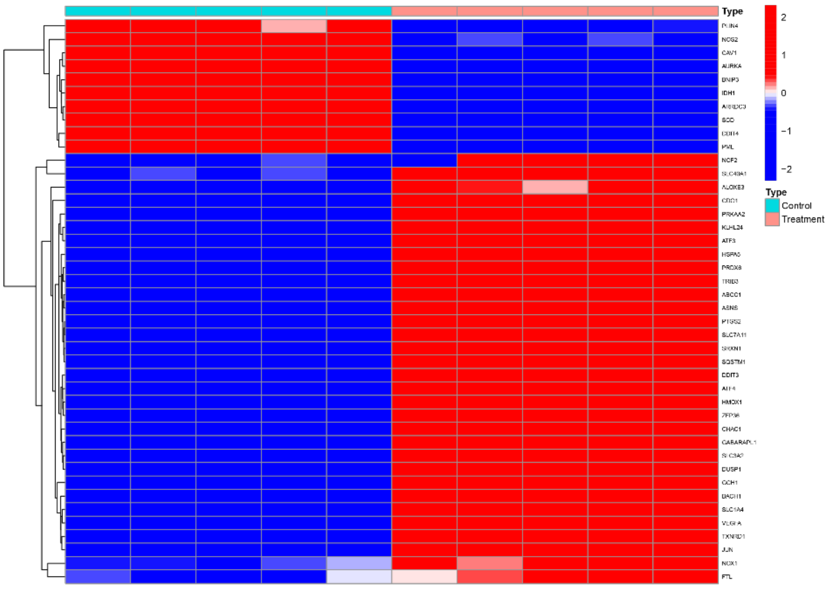


**Figure S30.** Heatmap of markers for ferroptosis selected from the mRNA sequencing data after OUSMNs treatment.

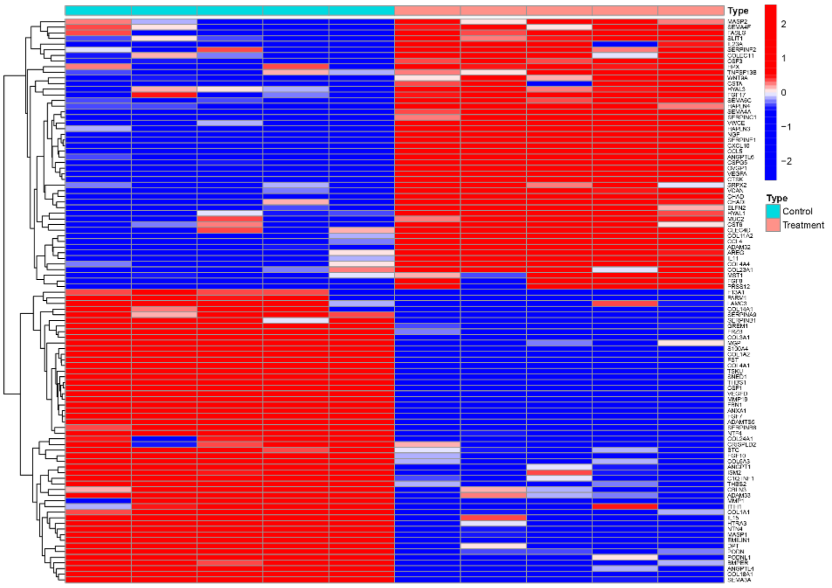


**Figure S31.** Heatmap of markers for extracellular matrix (ECM) deposition selected from the mRNA sequencing data after OUSMNs treatment.

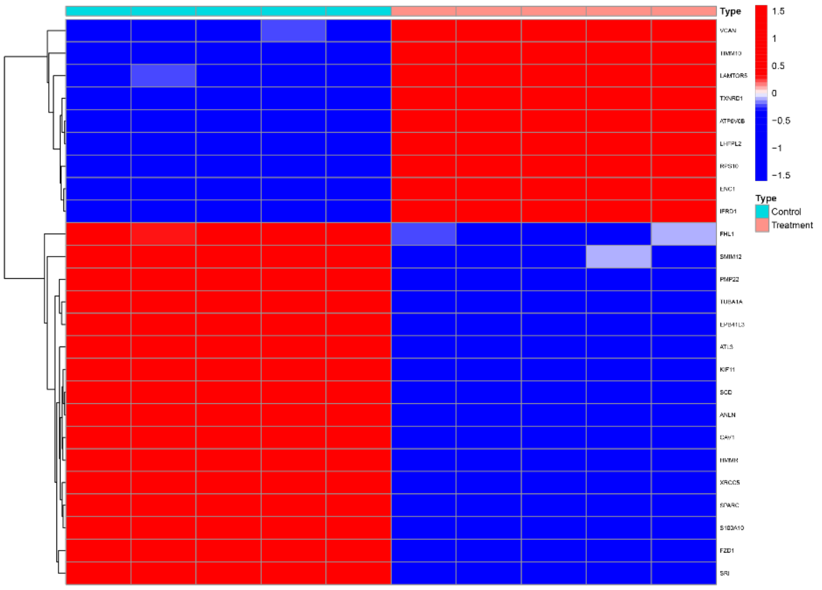


**Figure S32.** Heatmap of markers for epithelial-mesenchymal transition (EMT) process selected from the mRNA sequencing data after OUSMNs treatment.

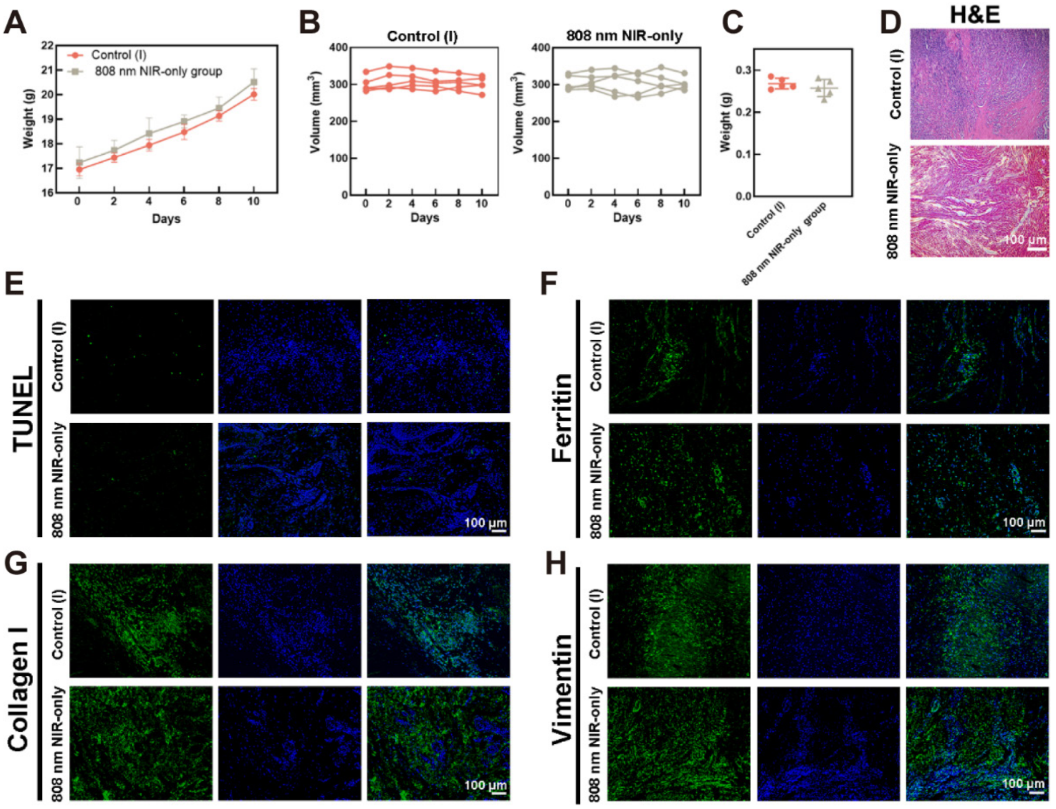


**Figure S33.**The effect of 808 nm NIR-only irradiation on a subcutaneous keloid xenograft model in nude mice. (A) Body weight variations in nude mice treated with NIR-only irradiation. (B) The growth curves of keloid transplantations in the control group and 808 nm NIR-only irradiation group. (C) Weight of keloid grafts in the control group and 808 nm NIR-only irradiation group. Histological observation of the keloid grafts with staining of (D) H&E, (E) TUNEL, (F) Ferritin, (G) Collagen I, and (H) Vimentin in the control group and 808 nm NIR-only irradiation group.

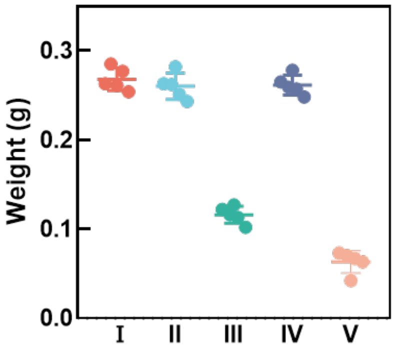


**Figure S34.** Weight of keloid grafts in groups I- IV.

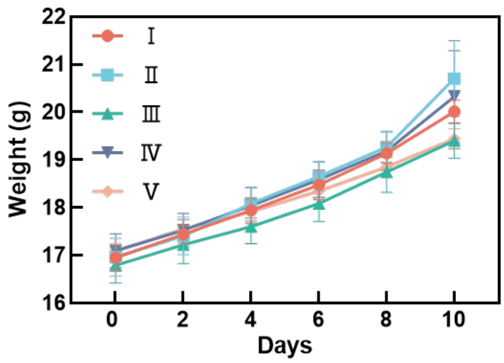


**Figure S35.** Body weight variations in nude mice treated with groups I- IV.

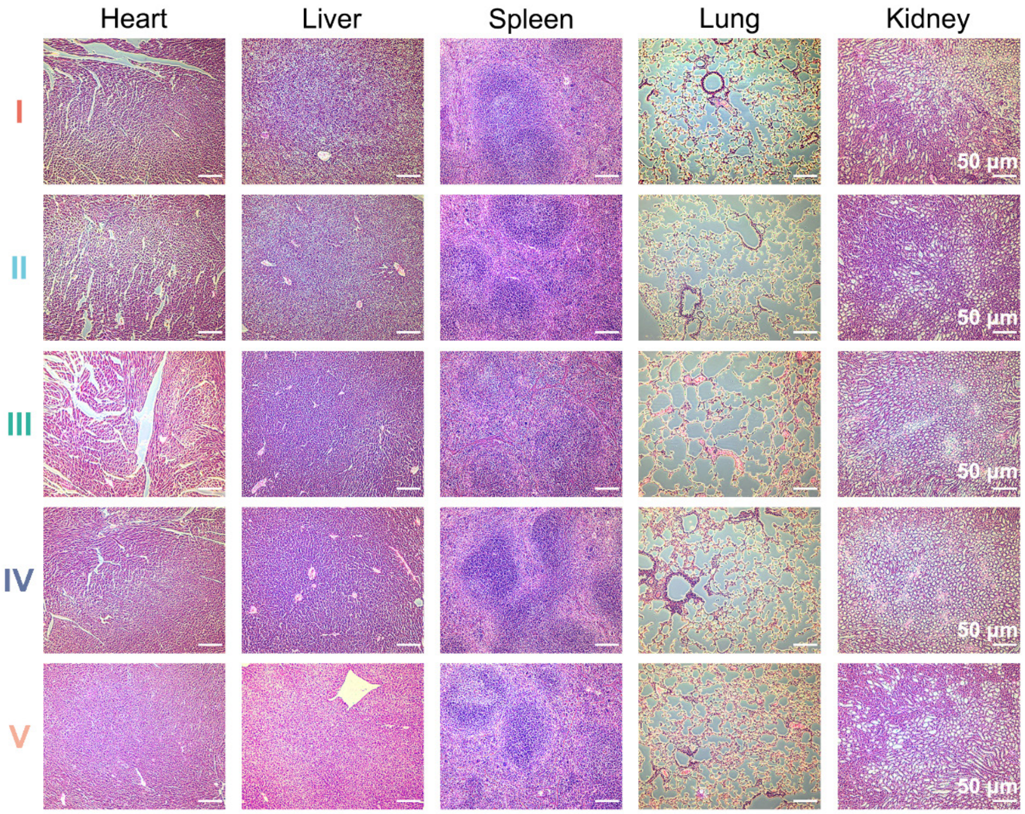


**Figure S36.** H&E-stained images of vital organs in nude mice with groups I- IV.

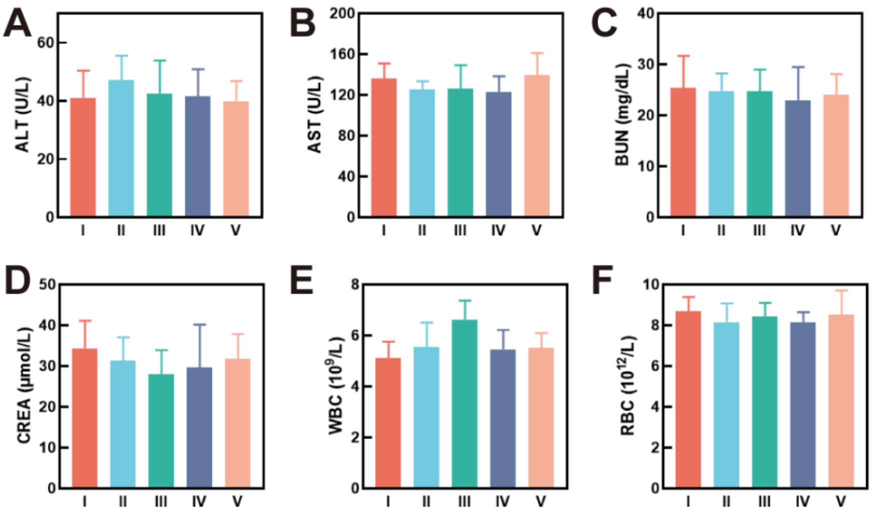


**Figure S37.** Assess the liver and kidney functions of nude mice treated with groups I-IV.

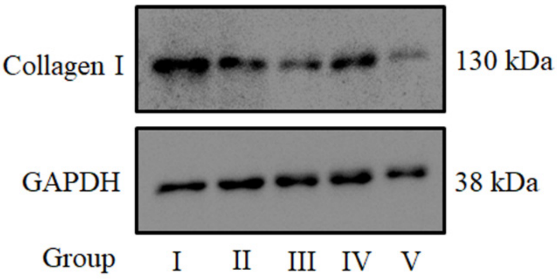


**Figures S38.** Western blot analysis was conducted to measure Collagen I expression protein level.
